# Origin of tuberculosis in the Paleolithic predicts unprecedented population growth and female resistance

**DOI:** 10.1038/s41598-019-56769-1

**Published:** 2020-01-08

**Authors:** Pere-Joan Cardona, Martí Català, Clara Prats

**Affiliations:** 1 Unitat de Tuberculosi Experimental, Institut de Recerca Germans Trias i Pujol (IGTP), Universitat Autònoma de Barcelona, CIBERES, Badalona, Catalonia Spain; 2 Comparative Medicine and Bioimage Centre of Catalonia (CMCiB). Fundació Institut d’Investigació en Ciències de la Salut Germans Trias i Pujol, Badalona, Catalonia Spain; 3 Escola Superior d’Agricultura de Barcelona, Departament de Física, Universitat Politècnica de Catalunya (UPC)-BarcelonaTech, Castelldefels, Catalonia Spain

**Keywords:** Computational biology and bioinformatics, Ecology, Evolution, Microbiology

## Abstract

Current data estimate the origin of *Mycobacterium tuberculosis* complex (MtbC) infection around 73,000 years before the common era (BCE), and its evolution to “modern” lineages around 46,000 BCE. Being MtbC a major killer of humanity, the question is how both species could persist. To answer this question, we have developed two new epidemiological models (SEIR type), adapted to sex dimorphism and comparing coinfection and superinfection for different MtbC lineages. We have attributed a higher resistance/tolerance to females to explain the lower incidence noted in this sex, a better health status in the Paleolithic compared to the Neolithic, and a higher dissemination of “modern” lineages compared to “ancient” ones. Our findings show the extraordinary impact caused by “modern” lineages, provoking the extinction of the groups infected. This could only be overcomed by an unprecedented population increase (x20 times in 100 years) and helped with the protection generated by previous infection with “ancient” lineages. Our findings also suggest a key role of female resistance against MtbC. This data obliges us to rethink the growth population parameters in the Paleolithic, which is crucial to understanding the survival of both MtbC and humans, and to decipher the nature of human female resistance against TB.

## Introduction

Tuberculosis (TB) is a major threat to humankind. Indeed, it has been estimated that this disease has caused 1,000,000,000 deaths in the last 200 years^1^. Despite the major efforts made to control it, including the world emergency declared by the WHO in 1993^2^, TB still is a challenge, causing 1.5 million deaths last year alone. In addition, the incidence of this disease is declining only slowly despite the considerable global efforts invested in trying to improve its prevention, diagnosis and treatment^3^. Several groups have attempted to determine the origin of *Mycobacterium tuberculosis* Complex (MtbC) in order to better understand its highly virulent nature. In contrast to the classic theory whereby it originated as a zoonotic infection, evolving from an ancestor of *Mycobacterium bovis*^4^, current data support the opposite. Thus, MtbC’s most recent common ancestor (MRCA) emerges around 73,000 years before the common era (BCE), originating specifically in the so-called anatomically modern humans (*H. sapiens*) from an environmental mycobacterium^5–7^. This is linked to the onset of controlled fire use, and thus smoke exposure and increased physical contact among individuals around the fire^8^. Interestingly, this MRCA differs from “ancient” and “modern” lineages in a context of low population densities around 70,000 and 46,000 BCE, respectively^6,7^. During this period, known as the Middle Paleolithic, the most populated continent (Africa) had sustained a human presence since roughly 2,000,000 BCE in a context of several glaciations and their very balanced lifestyle^9^. Organized into small tribes of around 50 individuals, these humans were nomadic hunter gatherers, had a good health status thanks to a lifestyle based on a varied diet, low work intensity and moderate exercise. This resulted in a life expectancy of around 33 years^10,11^. However, this population did not grow due to low birth rates and the difficulty in surviving up to the age of 15 (only 57%) as the cost of children was high^11^. Once they had reached the age of 15, 67% of these humans lived to an age of 45 or older^12^. This explains why, even in the context of a good lifestyle, the human population remained stable for hundreds of thousands of years, with a population growth of around 0.003%/year or less^13,14^. This scenario is radically different to the one observed during the increased growth population explosion linked to the Neolithic revolution, of around 0.1%/year^13^, when human activity became more farming-based, which led to a more sedentary life style and higher birth rates, but also a lower quality of life and increased mortality^10,15^.

In this context, the question remains as to how a devastating disease like TB did not eradicate humankind given the low population density of the Middle Paleolithic. We have also addressed this question by considering the particularities of the bifurcation that occurred 46,000 BCE with the onset of “modern” lineages after loss of the Tbd1 gene region^5^. Various studies have tried to discern the biological differences between these lineages and modern Mtb strains, using mainly *M. africanum* (lineages 5–7) and lineage 1 for the “ancient” strains and lineages 2–4 for the “modern” ones. Portevin *et al*.^16^ compared the innate response triggered in macrophages and dendritic cells by different strains from both lineages and concluded that “ancient” ones induce a higher pro-inflammatory profile when infecting macrophages and dendritic cells. These results have been interpreted as a virulence/immune response trade-off for “ancient” strains as bacilli are discovered early by the immune system and have less chance to progress, including extrapulmonary dissemination, which contrasts with the situation found for modern strains^17^. However, the current interpretation does not include this trade-off as this pro-inflammatory profile can, in fact, enhance the infiltration of neutrophils into Mtb-infected lesions, thus resulting in a better chance to quickly progress to active TB^18^. Bold *et al*.^19^ discovered a relevant characteristic of *M. africanum*, namely the larger size of the bacilli. This fact could indeed be a trade-off between progression to disease versus dissemination as it might hamper the production of small aerosols, which are best able to effectively reach the alveolar macrophages^20^, thus limiting the spread of these lineages.

Thus, the trade-off for “ancient” MtbC lineages is the higher probability of progression to active TB at a cost of being less able to disseminate through the population explaining why “ancient” lineages can only be found in certain geographical locations^21^. The fact that the appearance of “modern” lineages (46,000 BCE) coincided with a significant increase in the population (from 10^4^ to 10^6^ individuals) in Asia, while remaining constant in Africa (around 10^6^ people)^22,23^, indicates that some sort of population explosion occurred in Asia before the Neolithic, as proposed by several authors^24–26^, giving support to its dissemination.

The current interpretation of the coevolution between humankind and MtbC is based on the hypothesis that the mechanism of infection of MtbC was originally based mainly on the induction of latent infection, with a late progression towards active disease (i.e. prolonged latency) of more than a generation, with younger and more susceptible individuals subsequently becoming infected^27^. In accordance with these criteria, Zheng *et al*.^28^ adapted the treatment-free model of TB transmission^29,30^ to a population of 100 individuals. In their model, these authors did not consider the special risk of recovered TB cases subsequently going on to develop active disease again, which is at least seven-times higher than in latently infected individuals^31^, although they did consider the exogenous reinfection process. They concluded that maintaining the persistence of MtbC required a high progression to disease of up to 50%, which far exceeds the value of 5–10% considered nowadays^32^, justifying this by assuming a progressive increase in resistance acquired by humanity over time. The achievement of a considerable level of resistance in a community requires a large proportion of the population to be submitted to a mortality high enough to select innately resistant subjects, as shown by several authors^14,33,34^. The question is, could the Paleolithic population afford such a high progression rate?

From an eco-immunological viewpoint, host resistance reduces the parasite concentration (i.e. exploitation) below the optimum for the bacilli, thus leading to a counter-adaptation to enhance exploitation^35^. This could lead to a negative scenario for humans as parasites are better able to adapt due to the larger size of their populations and shorter generation times, which would lead to an “arms race co-evolution”^36^. This would lead to a theoretically indefinite escalation in resistance against exploitation, which would most probably defeat the host. In this regard, Bergelson^37^ proposed a sort of cyclical dynamic, with escalation followed by a reduction in competition^35^. Equally, there is another mechanism, namely tolerance, that can benefit both organisms. Tolerance is the ability to maintain fitness when hosting a high parasite concentration. Moreover, tolerance increases the fitness of the host, tends to be fixed, and at the same time allows persistence of the infection^38^. Overall, it appears that natural selection tends to favor combinations of resistance and tolerance (high tolerance and low resistance, or low tolerance and high resistance, or intermediate values of both), a correlation that has been demonstrated in plants^39^.

In light of these factors, we have developed a new SEIR model based on previous versions^28,29^ that includes known factors such as exogenous reinfection and the increased susceptibility to progression to TB in individuals who have already suffered TB disease and have recovered^31^. We have also included the constant bacillary expulsion, together with the decrease in immunity observed with time^40–42^, avoiding the concept of “once infected always infected and protected” proposed by Stead^43^, which is currently no longer valid^42,44^. In this model, we have considered the most recent data available concerning fast progression and reactivation determined for infection with “modern” lineages^45,46^, together with data on natural cure and mortality in untreated HIV-negative patients^47^. We have also taken into account the health conditions of humans in the Paleolithic and Neolithic, which might have affected changes in protection mechanisms, understood to be a combination of tolerance and resistance mechanisms. In addition, we have considered this mechanism to explain the different incidence in the two sexes (globally, 64% of cases are found in males and 36% in females)^3^. Indeed, the lower TB incidence in women is a matter of controversy as it has been attributed largely to cultural and socioeconomical inequalities against women^48^. However, this concept is currently being challenged, with the opposite, i.e. an inequity of health services among men, being claimed^49^. The idea of some type of natural protection in women due to biological mechanisms was first raised several years ago^50^.

Application of these concepts may help us to understand how “modern” MtbC lineages, with their current dissemination capacity, were able to persist until the present without eliminating humanity. In this regard, we have also established a co-infection model in order to study the replacement of “ancient” lineages with their “modern” counterparts found nowadays.

To the best of our knowledge, this is the first time that the concepts of tolerance/resistance and sex differences have been taken into consideration when trying to understand TB epidemics, in addition to co-infection with different lineages. Our findings should be taken into consideration in the current analysis of global epidemics, in which 1/3 of TB cases are not even identified. This is a paramount factor that should be addressed to stop the pandemic and to improve the survival of all subjects at high risk of developing TB.

## Results

### The irruption of “modern” lineages caused a dramatic impact in the Paleolithic

**Figure 1:**
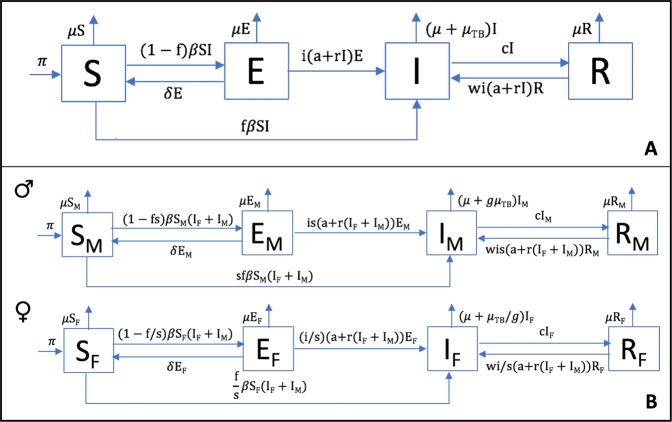
SEIR model. Each compartment refers to the set of individuals by disease. status: Susceptible, Exposed, Infected, Recovered. New-born individuals are assumed. susceptible. A TB infection can remain latent (**E**), or can directly develop into infectious. active TB (**I**). The latent TB infection can become active through endogenous reactivation or exogenous reinfection. Patients with active TB can naturally recover (**R**) becoming non-infectious. Latent infected persons (**I**) can drain the bacilli, lose the immunity and become susceptible (**S**). Recovered persons can relapse to active TB through endogenous reactivation or exogenous reinfection. Picture **A** shows the generic model, and picture **B** the one that considers the sexual dimorphism (TBOREX), where new-born entrance $\pi$ depends on the fertility of females.

**Figure 2:**
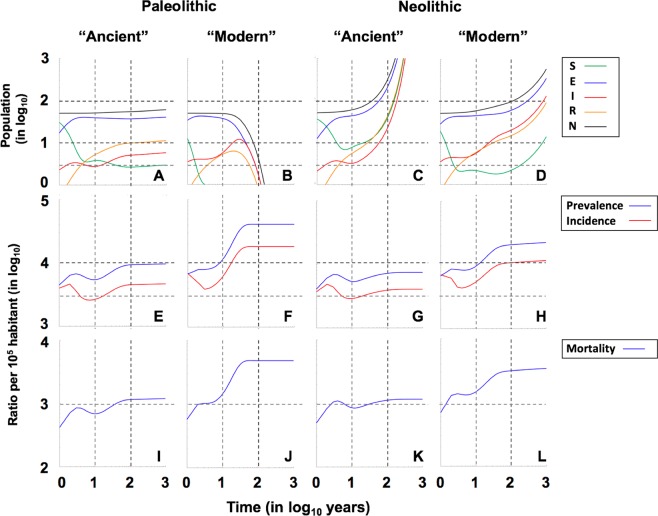
Evolution of the population in the continuous model TBOREX. Pictures show the projections for 1000 years in initial populations of 50 members where a unique male TB patient is included (I_o_ = 1). Evolution is drawn under Paleolithic (**A,B,E,F,I,J**) and Neolithic (**C**,**D,G,H,K,L**) life conditions, in relation to the MtbC lineage infection, “Ancient” (**A**,**E,I,C,G,K**) or “Modern” (**B**,**F,J,D,H,L**). Continuous lines in pictures **A** to **D** show the total population (**N**) (**black**), Non-infected susceptible (**S**) (**green**), Infected (**E**) (**blue**), TB patients Infectious (**I**) (**red**) and previous TB patients Recovered (**R**) (**orange**). Figure C is truncated as the levels grow exponentially to reach final outcomes of >10^9^ persons. Continuous lines in pictures **E** to **H** show the Incidence (**red**) and Prevalence (**blue**) of TB cases (**I**) per 100.000 inhabitants. Continuous lines in pictures **I** to **L** show the Mortality caused by TB cases per 100.000 inhabitants. Dotted lines represent reference values. We have considered 2 and 10 persons (population), 3.000 and 10.000 (incidence and prevalence), 300 and 1.000 (mortality) and 10 and 100 years (time).

We have designed a compartmental mathematical model (TBOREX) (TB, Origen and Sex) based on five differential equations to describe the dynamics of the evolution of MtbC infection in the population, based on previous models^28–30,51^ (Fig. 1 and Table 1). The standard scenario modelled is a human group of 50 persons in which a single infectious male is initially included. Figure 2 shows the progression of both MtbC lineages in the conditions of the Paleolithic and Neolithic periods.

**Table 1:** Parameters and references.

Parameter	Values		Sources
	Paleolithic	Neolithic	
Annual population growth rate	1%	2.6%	^13,26,52^
Number of births per fertile woman	2.8	4.7	
Number of births per fertile woman/year ($\lambda$)	0.078	0.128	
Natural mortality/year ($\mu$)	1/33	1/26.5	^73,74^
Mortality/year caused by TB ($\mu$_TB_)	0.12	0.15	^47^
Infected people per case/year (e)	10(A)/20(M)		^67^
Fast progression (f)	0.099(A)/0.0825(M)	0.1238(A)/0.1031(M)	^45^
Reactivation from infection (a)	f · 0.3		
Bacillary drainage and immunity reduction ($\delta$)	0.1-a-r		^40,41^
Reduced progression due to immunity (i)	0.1		^69^
TB natural cure (c)	0.33		^47^
Increased progression in Recovered (w)	7		^31^
Male/Female TB tolerance (s) (g)	55/45		^50^

**Figure 3:**
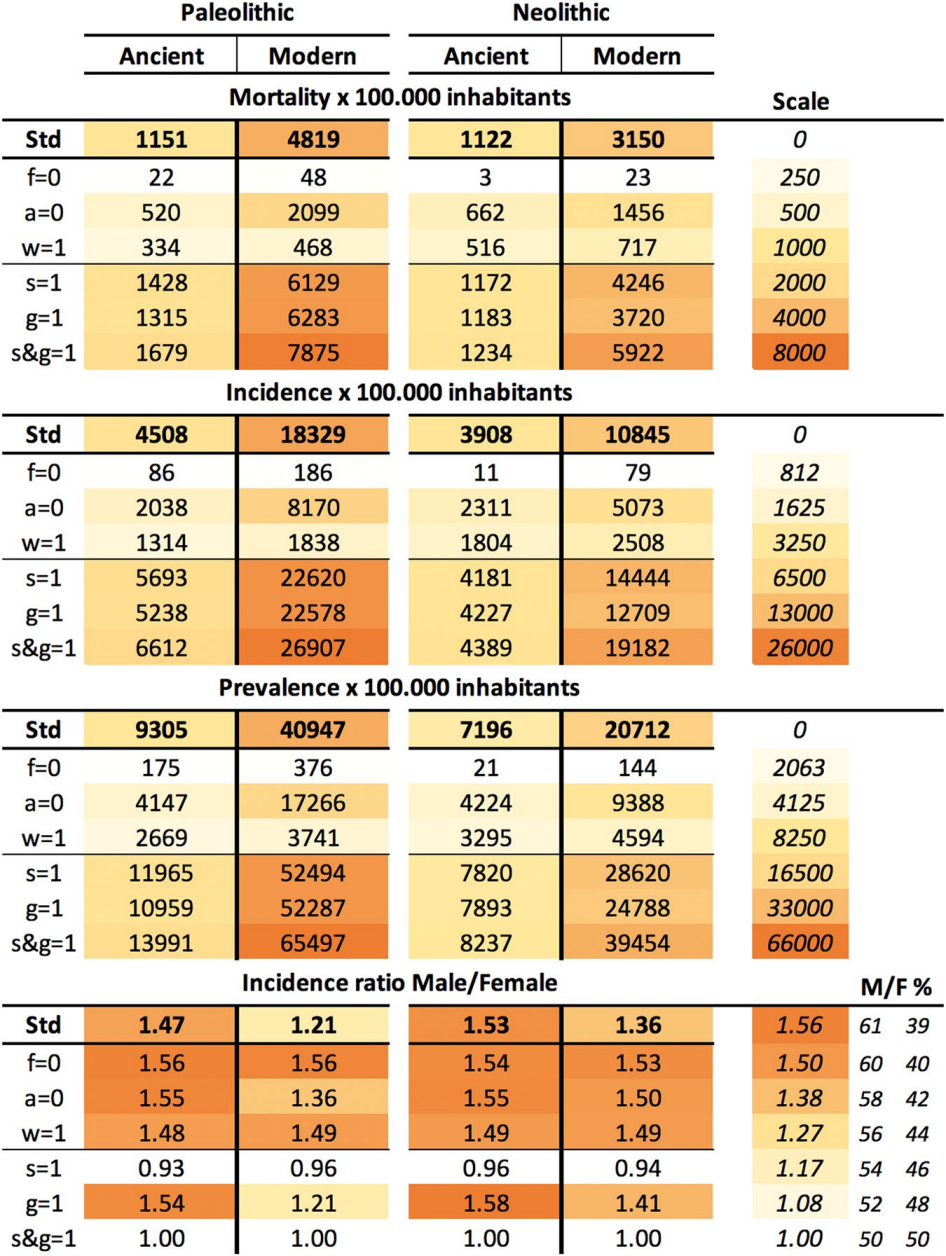
End values in the continuous model TBOREX. Influence of progression factors. Heatmap of the projections for 100 years in initial populations of 50 members where a unique male TB patient is included (I_o_  =  1). Cells are colored according the following rule: minimal (white), and maximal (orange). A color legend has been added per each dataset as a reference.

**Figure 4:**
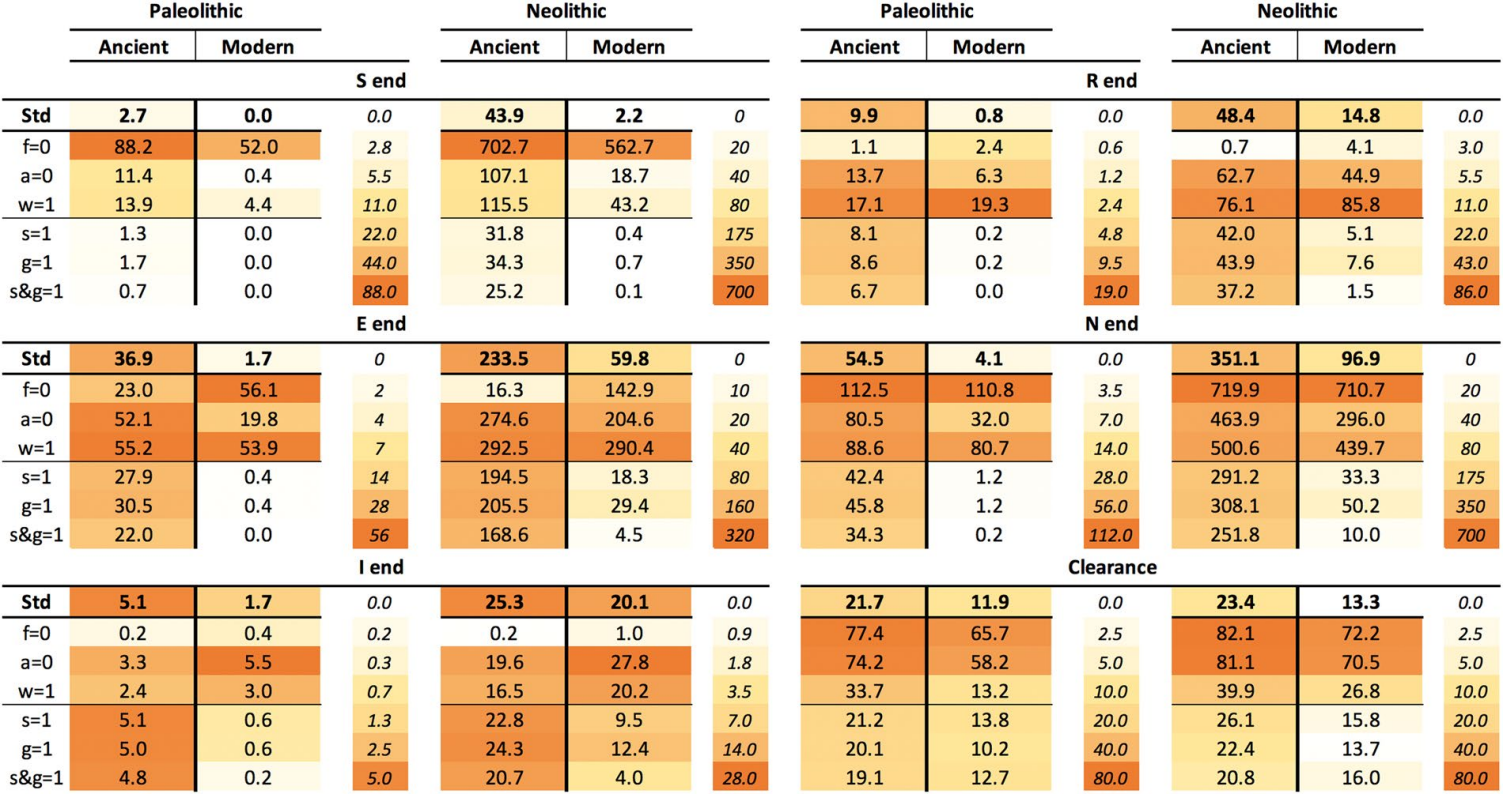
End values and clearance in the TBOREX model simulation. Influence of progression factors. Heatmap of the end values for the projections for 100 years in initial populations of 50 members where a unique male TB patient is included (I_o_  =  1). Cells are colored according the following rule: minimal **(white)** and maximal (orange). A color legend has been added per each dataset as a reference.

In the case of a Paleolithic community with the “ancient” strain, the logarithmic scale shows two periods. The first of these, known as the “attack” period, occurs during the first 10 years after one infectious case enters the communities, thus representing initial dissemination. This phase is characterized by a sudden increase in the number of exposed and infectious cases, which is linked to a decrease in the susceptible population (Fig. 2A). The global population remains stable at a cost of a high annual mortality (Fig. 2I), reaching levels of 1000 deaths per 100,000 inhabitants, together with an initial reduction in incidence and prevalence (Fig. 1E). A second wave then appears, with this wave following a growth that stabilizes the epidemic 80 years post-challenge (Fig. 2E) with a mortality slightly higher than 1000 deaths/100,000 inhabitants and an incidence and prevalence of 4508 and 9305 per 100.000 inhabitants, respectively (Figs. 2E and 3). Interestingly, there is a constant growth in the recovered population, with a “stable” period being reached at 100 years post-challenge. The susceptible compartment undergoes a negative progression that stops with a decrease in incidence, subsequently increasing slightly until the infectious compartment increase again, finally stabilizing at 5.1 cases (Fig. 4).

Infection with a “modern” lineage, with a much higher dissemination power, rapidly extinguishes the susceptible compartment under Paleolithic conditions (in three years), as shown in Fig. 2B. Infectious cases reach a plateau, which is followed by a parabola-like kinetic that peaks at more than 10 cases (Fig. 2B) as a result of the rapid progression of infected and recovered cases. This results in a decline in the population, after stabilization of the epidemic, at an extremely high incidence of 18,329 cases/100,000 inhabitants and a mortality of 4819 (Fig. 2F,J, and 3). Note that, under Neolithic conditions, the total population grows extremely rapidly during infection with “ancient” lineages, up to 351 persons, thus multiplying sevenfold in 100 years (Fig. 2C). In contrast, for a “modern” infection, the population roughly doubles in the same period (Fig. 2D).

### Higher population growth in the Neolithic allows the persistence of “modern” lineages

When analysing the Neolithic scenario, it appears that the first phase occurs as in the Paleolithic (Fig. 2), except for the higher number of susceptible individuals as a consequence of the higher population growth. In the following phase, between 10 and 100 years, growth is exponential in both cases, especially during infection with the “ancient” lineage. However, this is a non-realistic scenario as, after 1000 years, the population reaches more than a billion if the birth rate is not limited by a logistical growth with a specific carrying capacity. In addition, given the higher dissemination capacity and lower clearance of “modern” lineages, it can be assumed that these lineages displaced “ancient” ones and markedly restrained the potential growth of the Neolithic population. This will be analysed below, with the co-infection model. In this case, the incidence of infectious persons increases to a significant 10% of the population (Fig. 2H), with a slightly increasing trend.

### “Modern” lineages are better able to persist in the population

**Figure 5:**
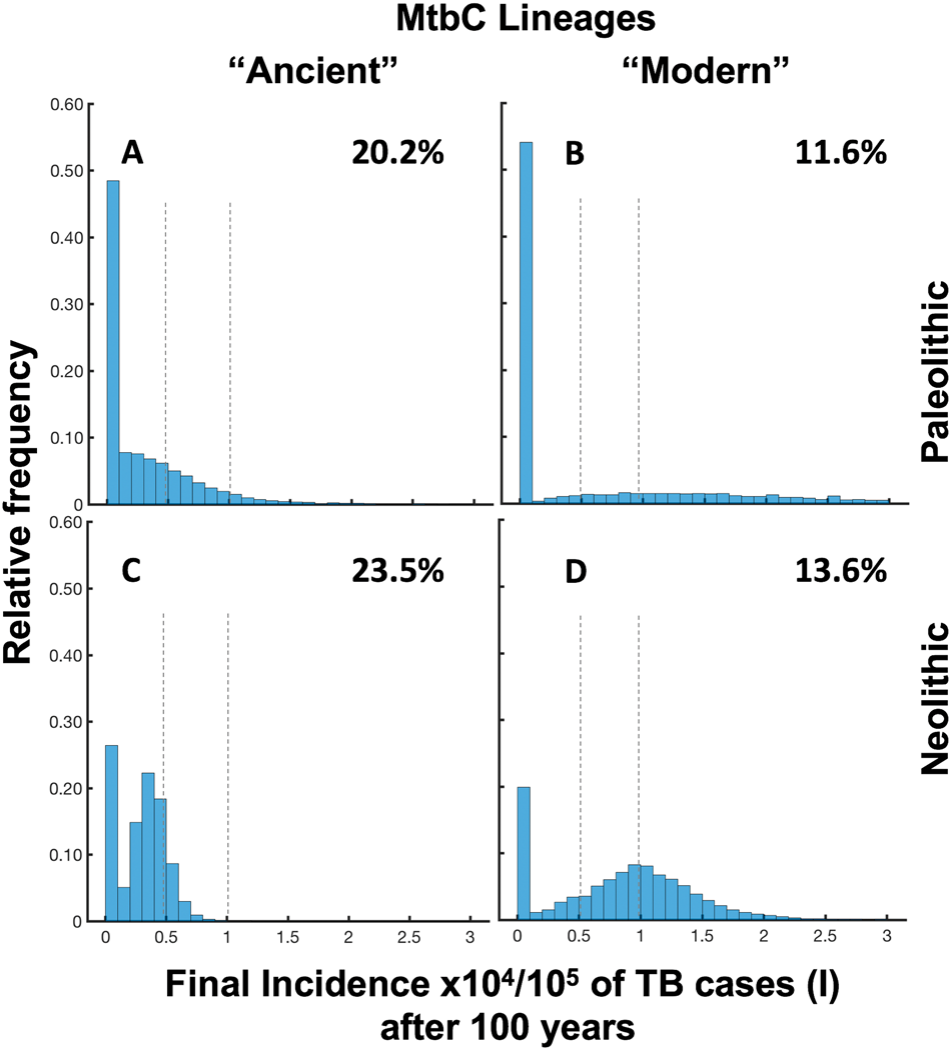
Distribution of final incidence per 100.000 habitants of TB cases in the discrete model TBOREX and infection clearance (absence of EIR). We have run the model 10.000 times. Pictures show the projections for 100 years in initial populations of 50 members where a unique male TB patient is included (I_o_ = 1). Evolution is drawn under Neolithic life conditions, in relation to the MtbC lineage infection, “Ancient” (**A,C**) or “Modern” (**B,D**). Percentage of clearance is written in the upper right. Dotted lines represent reference values, 5.000 and 10.000 TB cases (**I**)/100.000 h.

The continuous resolution of the model provides a unique global view of the progression which, unfortunately, does not fully reflect reality as we are working with very low numbers. In addition, the continuous resolution of the model’s equations allows the average dynamics of the system to be observed but does not account for the inherent variability between different communities due to their limited size. In particular, a number of people between 0 and 1 in the infectious compartment is halfway between completely different situations that correspond to the absence (0) or presence (1) of individual people in this compartment. This is why we decided to work with the TBOREX discrete resolution, where values are transformed into natural numbers using random numbers, as detailed above (Supplementary Fig. 2). Figure 5 shows the percentage of runs in which the TB-affected compartments (E, I and R) disappear after running the program 10,000 times. Indeed, this simulation series emulates the behaviour of epidemics in 10,000 independent communities using the same model and parameters, but with a certain degree of randomness to account for inherent variability. The results reflect how “modern” lineages are cleared from communities less often (almost half) than the “ancient” ones in periods of 100 years, thus confirming that a higher capacity for dissemination is a key factor for persistence of these epidemics. The distribution of the final TB incidence found for the Paleolithic scenario, before extinction of the community due to a “modern” lineage infection, is of special interest given the wide range of possible final incidences.

### Fast progression, immunity, latency and higher susceptibility in recovered subjects are required to maintain epidemics

**Figure 6:**
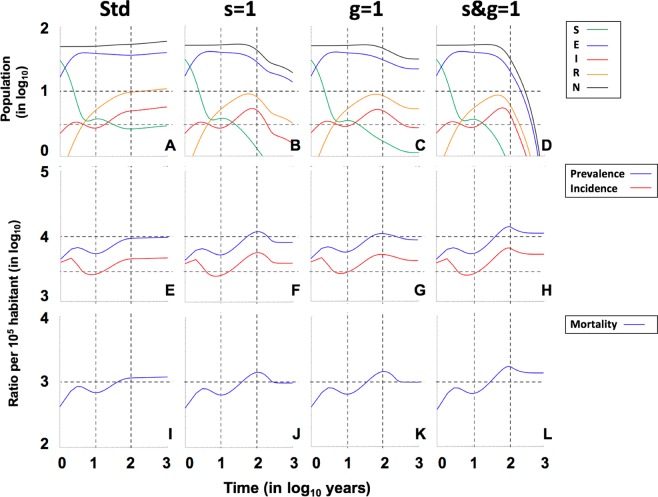
Role of the female tolerance in the evolution of “Ancient” lineages in the Paleolithic. Pictures show the projections for 1000 years in initial populations of 50 members where a unique male TB patient is included (I_o_ = 1), using continuous resolution of TBOREX model. Evolution is drawn under standard (**Std**) conditions (**A,E,I**), neutralizing the factor”**s**” (**B,F,J**), “**g**” (**C,G,K**) or both, “**s&g**” (**D,H,L**). Continuous lines in pictures **A** to **D** show the total population (**N**) (**black**), Non-infected susceptible (**S**) (**green**), Infected (**E**) (**blue**), TB patients Infectious (**I**) (**red**) and previous TB patients Recovered (**R**) (**orange**). Continuous lines in pictures **E** to **H** show the Incidence (**red**) and Prevalence (**blue**) of TB cases (**I**) per 100.000 inhabitants. Continuous lines in pictures **I** to **L** show the Mortality caused by TB cases per 100.000 inhabitants. Dotted lines represent reference values. We have considered 2 and 10 persons (population), 3.000 and 10.000 (incidence and prevalence), 1.000 (mortality) and 10 and 100 years (time).

**Figure 7:**
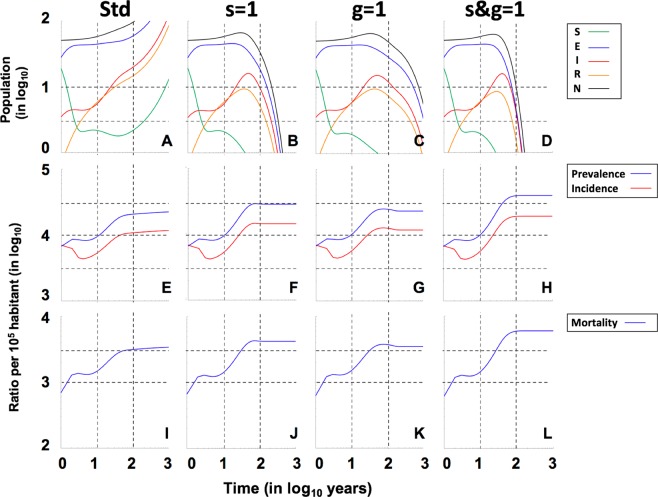
Role of the female tolerance in the evolution of “Modern” lineages in the Neolithic. Pictures show the projections for 1000 years in initial populations of 50 members where a unique male TB patient is included (I_o_  =  1), using continuous resolution of TBOREX model. Evolution is drawn under standard (**Std**) conditions (**A,E,I**), neutralizing the factor”**s**” (**B,F,J**), “**g**” (**C,G,K**) or both, “**s&g**” (**D,H,L**). Continuous lines in pictures **A** to **D** show the total population (**N**) (**black**), Non-infected susceptible (**S**) (**green**), Infected (**E**) (**blue**), TB patients Infectious (**I**) (**red**) and previous TB patients Recovered (**R**) (**orange**). Figure **A** is truncated as the levels grow exponentially to reach final outcomes of >500 persons after 100 years. Continuous lines in pictures **E** to **H** show the Incidence (**red**) and Prevalence (**blue**) of TB cases (**I**) per 100.000 inhabitants. Continuous lines in pictures **I** to **L** show the Mortality caused by TB cases per 100.000 inhabitants. Dotted lines represent reference values. We have considered 2 and 10 persons (population), 3.000 and 10.000 (incidence and prevalence), 1.000 (mortality) and 10 and 100 years (time).

Figures 6 and 7, and Supplementary Figs 3–6, analyse the impact of neutralizing different factors that are intrinsic to the nature of MtbC infection, such as fast progression (*f*) and reactivation from latent infection (*a*), since these factors have been used to explain the persistence of MtbC^27,28^. The results show the extreme importance of fast progression, without which epidemics disappear in all cases due to an almost 100-fold reduction in incidence (Supplementary Figs 3–6). Neutralization of endogenous reactivation (*a*) seems to have a lower impact in terms of incidence, prevalence and mortality (around twofold), but, curiously, when looking at the clearance factor, it appears to have a similar impact (increase of about 3.5-fold) as the neutralization of fast progression (Fig. 4). This illustrates that both factors are essential for the maintenance of TB epidemics.

The second most important factor is the higher susceptibility in recovered cases (*w*), neutralization of which reduces the incidence by three- and 10-fold in “ancient” and “modern” lineages, respectively (Supplementary Figs 3–6). This factor has a lower impact on the clearance of epidemics (less than twofold) (Fig. 4).

**Figure 8:**
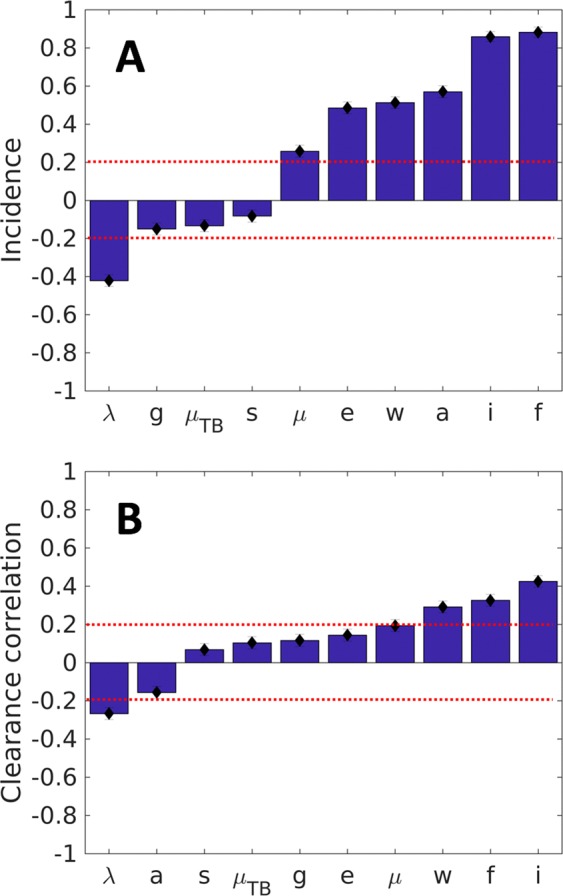
Sensitivity analysis. Partial Rank Correlation Coefficient on the TBOREX discrete model analyzing 1000 simulations. Parameters: birth rate ($\lambda$), natural mortality ($\mu$), mortality caused by TB ($\mu$_TB_), fast progression (*f*) and reactivation factor (*a*), number of infections caused by a patient (*e*), reactivation factor in recovered (w), tolerance to progression to disease (s) and to disease (g), and immunity (i). Picture A shows the influence of each parameter in the incidence and B clearance of the infection. Dotted red lines show the variation of a 20%.

The sensitivity analysis confirmed this view (Fig. 8 and Table 2). In this case we have also added immunity (*i*), an increase in which (and thus decrease in the protection conferred by immunity) is an important factor for maintaining the incidence, as is the increase in natural mortality (*μ*). An increase in both these factors is also related to the increase in clearance (Fig. 8B). In contrast, the birth rate (*λ*) works in the opposite way by decreasing both incidence and clearance.

**Table 2:** Sensitivity analysis.

Parameter	Paleolithic value	Neolithic value	Sensitivity analysis range
$\lambda$	0.078	0.128	[0.07 0.13]
$\mu$	0.03030	0.03846	[0.0286 0.04]
${\mu }_{TB}$	0.12	0.15	[0.1 0.17]
$e$	10	20	[10 20]
$f$	0.099(A)/0.0825(M)	0.1238(A)/0.1031(M)	[0 0.13]
$i$	0.1	0.1	[0.05 0.5]
$w$	7	7	[1 7]
$a$	0.0297(A)/0.02475(M)	0.03714(A)/0.03093(M)	[0 0.038]
$g$	1.2222	1.2222	[1 1.4]
$s$	1.2222	1.2222	[1 1.4]

The importance of these factors changes with time, as can be seen from Supplementary Figure 7. Focusing on the TB-related classes (E, I and R), in the attack phase (i.e., the first 10 years), fast growth (*f*) and number of contacts (*e*) have particular relevance, subsequently becoming irrelevant once the epidemic has stabilized. Reactivation (*a*) also shows a similar correlation pattern in the particular case of infectious subjects. Birth rate (*λ*) evolves from a negative correlation at the beginning (i.e., the higher the birth rate the lower the number of infected/infectious/recovered) to a positive correlation in the final period (i.e., the higher the birth rate the higher the number of infected/infectious/recovered). From a global population perspective, the highest positive correlation is found with birth rate, whereas the death rate provides a high negative correlation, as expected. The fast progression parameter is also important but, in contrast to the TB-related classes, the correlation in this case is negative.

### Female resistance is key to understanding the co-evolution of MtbC and humans

Female protection merits a deeper analysis. Neutralization of both “g” and “s” factors (by giving a value of 1) appears to have not a marked impact in neither the endpoint values (Figs. 3 and 4) nor the sensitivity analysis (Fig. 8 and Suppl. Figure 7). This is because these analyses are based in projections for 100 years. On the contrary, the impact in the “critical” scenarios, i.e. the Paleolithic period infected with “ancient” lineage (Fig. 6) and the Neolithic infected with the “modern” one (Fig. 7), based in projections for 1000 years, is sufficient to change a persistent infection to elimination, together with the host.

Figure 3 shows the impact of these factors on the male/female incidence ratio. It appears that neutralization of the factor “s” (i.e. resistance), which results in equal sex progression to disease, is key as match up the male/female incidence at a proportion of around 50/50. On the contrary, neutralization of tolerance to the disease (g) has a low impact, keeping the ratio from 55/45 to 63/37 depending on the scenario, roughly like with the presence of both protection mechanisms (Std). This analysis precludes that resistance to disease (s) alone is able to explain the difference in incidence based on the sexual dimorphism.

**Figure 9:**
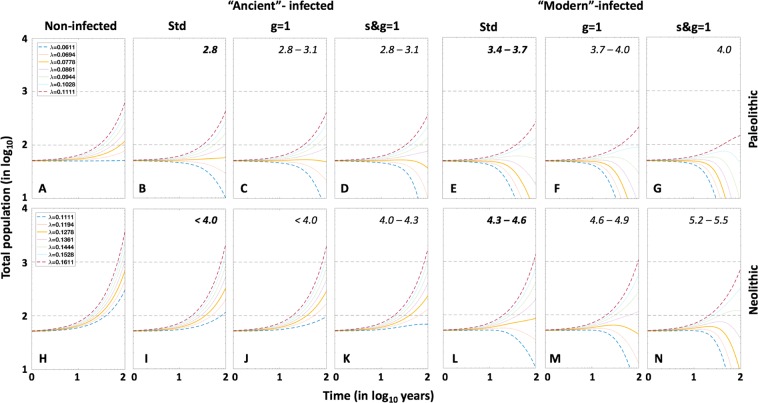
Natality and the evolution of total population. Influence of sex and strain. Evolution of total population, using continuous resolution of TBOREX model, with different natality indexes in TB-free communities (Non-infected), standard conditions (Std), neutralizing factor g (g = 1) and neutralizing factors g and s (s&g = 1). Continuous lines show the dynamics with the natality values used in the model (0.0778 in Paleolithic conditions and 0.1278 in Neolithic).

Figure 9 and Suppl. Table 2 illustrates the importance of these parameters in terms of demography in a projection of 100 years. In this figure we analyse the impact of female protection on population growth. The standard simulation (Std) conferred both resistance and tolerance to females by assuming a value of 45/55 for progression to disease (s) and TB mortality *μ*_TB_ (g), respectively (Std). We subsequently adjusted the birth rate (*λ*) to ensure the viability of the human population under the two “critical” scenarios, namely the Paleolithic and Neolithic with epidemics caused by the “ancient” and “modern” lineages, respectively. Without this protection, the birth ratio should increase from 2.8 to 2.8–3.1 births/fertile female in the Paleolithic and from 4.3–4.6 to 5.2–5.5 in the Neolithic, thus causing an approximate increase of 10% and 20% in birth rates, respectively. When considering only the resistance (s) in females, the impact is the same in the Paleolithic, but decreases to 7% in the Neolithic. Not to mention the impact when comparing with a non-infected population, where the number of births/fertile female could be as low as 2.2 births to keep a steady growth in the Paleolithic and 4 births to have an exponential growth and multiply x20 times the population in 100 years.

### “Modern” lineages replace “ancient” ones

**Figure 10:**
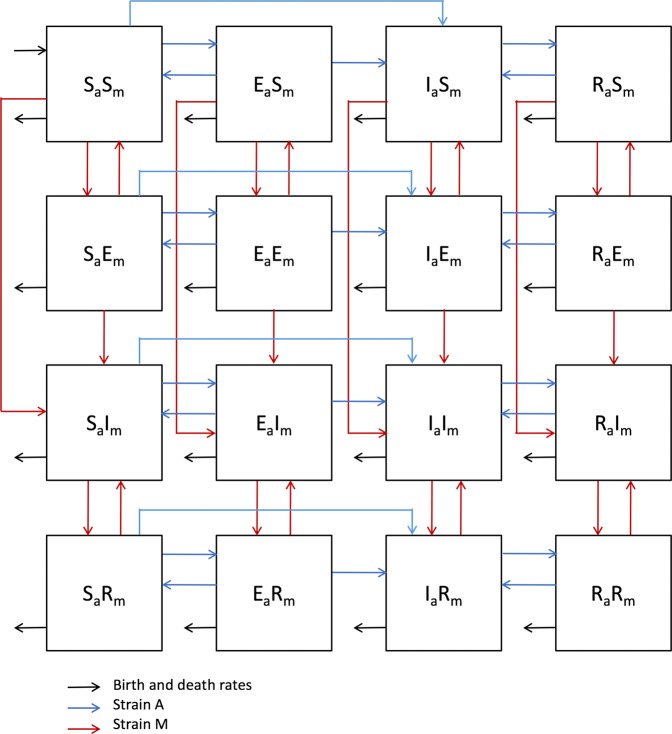
The Coinfection model. Each compartment refers to the set of individuals by disease status with regards to “ancient” (sub index a) and “modern” (sub index m) strains: Susceptible (**S**), Exposed (**E**), Infected (**I**), Recovered (**R**). New-born individuals are assumed susceptible. Color of arrows and subheadings refers to the evolution of the infection of each strain, corresponding to “ancient” lineage (**a**) (**blue**) and “modern” lineage (m) (**red**). The evolution of the coinfection causes not only the corresponding EIR population but also coinfection, where **E**_**a**_**E**_**m**_ represents a person with a latent coinfection, **I**_**a**_**E**_**m**_ an Infectious person with an “ancient” strain, latently infected with a “modern” strain, **E**_**a**_**I**_**m**_ the reverse, etc.

**Figure 11:**
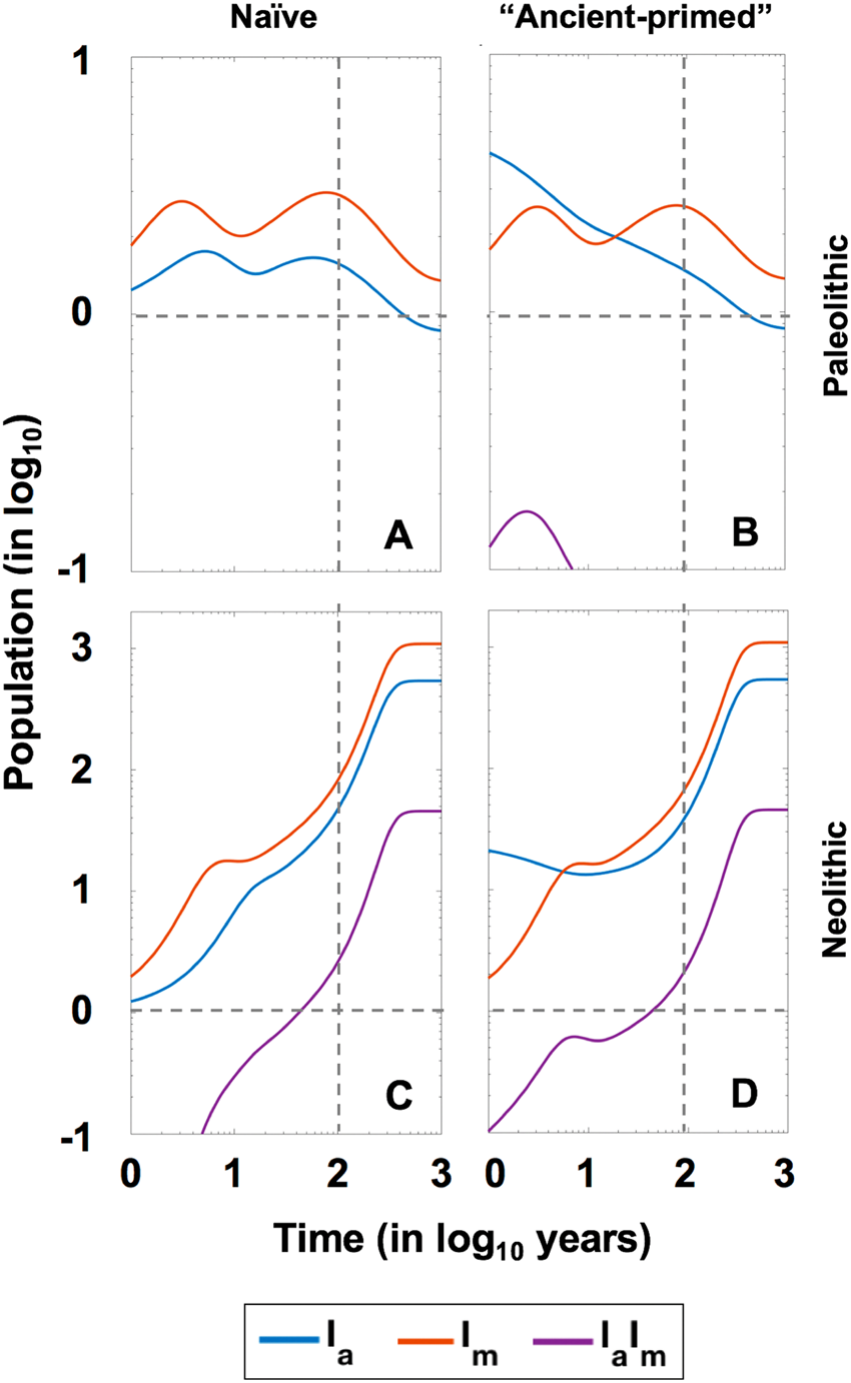
The replacement of “ancient” lineages by “modern” ones in a continuous coinfection model. Pictures show the evolution of the coinfection in four different circumstances, using the continuous coinfection model. Left quadrants show the evolution of the coinfection in the context of a “naive” population with two infectious males, one from each lineage, in the periods of Paleolithic (**A**) and Neolithic (**C**). Right quadrants show the evolution of the coinfection in the context of a community where infection with an “ancient” lineage has remained stable for 100 years and one “modern” lineage infected male is introduced, in the periods of Paleolithic (**B**) and Neolithic (**D**).

**Figure 12:**
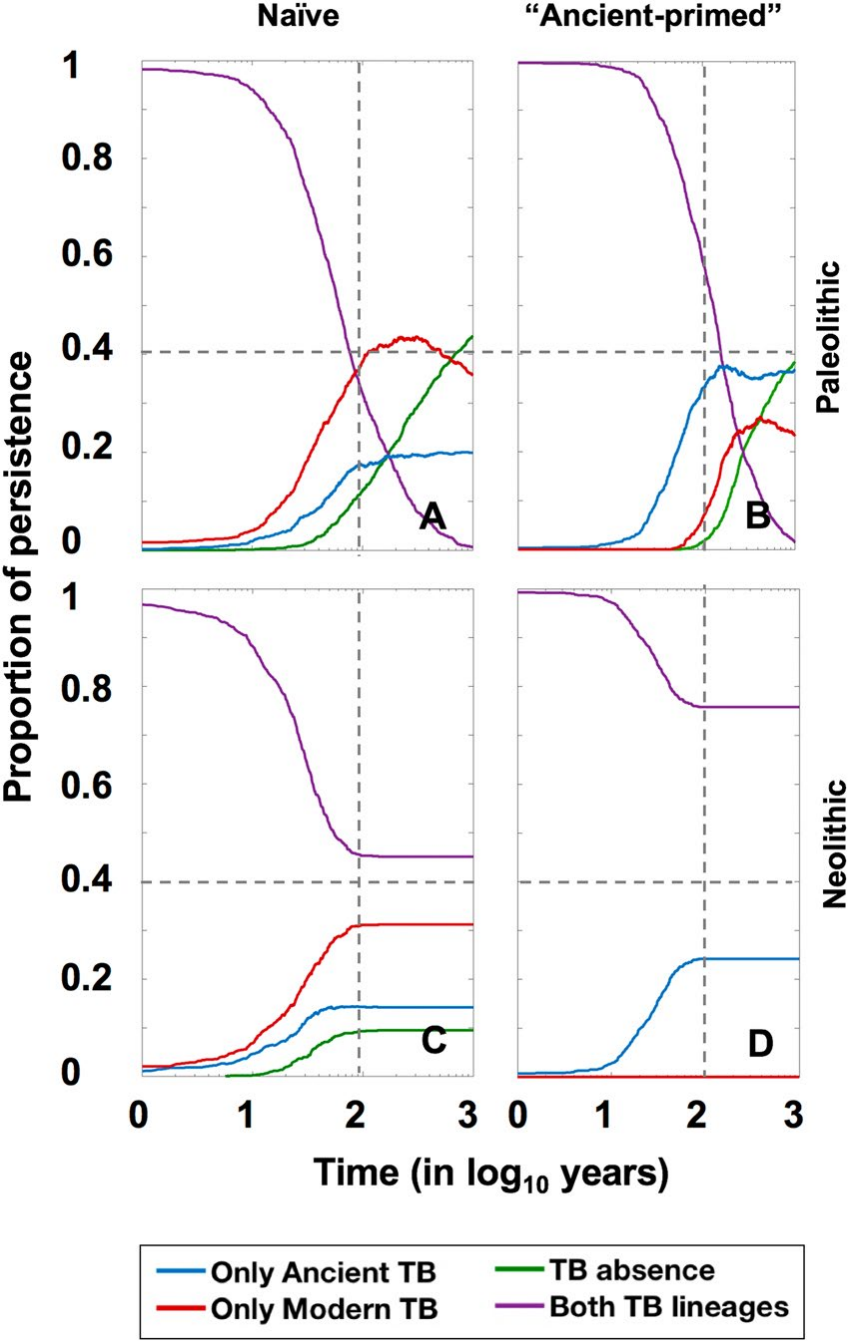
The replacement of “ancient” lineages by “modern” ones in a discrete coinfection model. Pictures show the persistence of the coinfection in four different circumstances, using the discrete coinfection model. 10000 independent repetitions of each scenario are run and the proportion of communities with presence of both strains (purple), only ancient (blue), only modern (red) or none of them (green) along the time are shown. Left quadrants show the persistence in the context of a “naive” population with two infectious males, one from each lineage, in the periods of Paleolithic (**A**) and Neolithic (**C**). Right quadrants show the persistence in the context of a community where infection with an “ancient” lineage has remained stable for 100 years and one “modern” lineage infected male is introduced, in the periods of Paleolithic (**B**) and Neolithic (**D**).

Looking at previous results on the incidence of infection, it appears that if a population is infected with both “modern” and “ancient” lineages, the former will predominate due its higher dissemination capacity. We have studied this aspect by building a new model in which we have considered all the coinfection circumstances (Fig. 10). As such, we designed two different scenarios. The simplest scenario initially includes two infectious males, one from each lineage, in a naïve population. The second scenario includes an infectious person carrying a “modern” lineage in the context of a “primed” community where infection with an “ancient” lineage has remained stable after 100 years (Figs. 11 and 12), which seems more realistic considering that “ancient” lineages were the only ones present for a period of more than 20,000 years.

The results in the continuous model show how, in naïve communities, “modern” lineages rapidly become predominant in the Paleolithic (Fig. 11), with the “ancient” one disappearing when the population experiences a decline (Suppl. Figure 8). In the case of a “primed” population, “ancient” lineages protect against the entrance of “modern” ones, which become predominant 20 years after the appearance thereof. In the Neolithic, the protection of “ancient” lineages lasts for seven years. Both lineages coexist, as we have seen in the Paleolithic, but in this case, thanks to the continuous population growth, the “ancient” lineages do not disappear in any case, even though the “modern” lineage has twice the number of cases (1000). In this case, it is noteworthy the slight presence of some cases (40) of coinfection (I_a_I_m_) detected in both cases, whereas there is no or only a very residual presence of coinfection under Paleolithic conditions.

Interestingly, when studying the ability of both lineages to persist after analysing the discrete model (Fig. 12), the scenario is different in the case of “primed” populations. In the Paleolithic, “ancient” lineages are able to remain predominant over the “modern” ones (37 vs 23%), with 2% of cases involving both strains and 38% of communities exhibiting global TB clearance. Furthermore, in the Neolithic, the most predominant form after 1000 years is coinfection (76%) followed by the “ancient” lineage (24%), whereas cases with only the “modern” lineage disappear. This scenario changes drastically when the initial conditions are modified. For instance, the entrance of five TB cases with “modern” lineages in a “primed” population leads to a scenario in which the “modern” lineage becomes predominant under Paleolithic conditions (38% of only “modern” to 19% of only “ancient”), whereas 100% of Neolithic communities maintain the presence of both strains after 1000 years (Suppl. Figure 9). These findings are due to two factors: (i) the “ancient” strain is very well established in Neolithic communities, which are larger than Paleolithic ones and therefore prevent the clearance of this strain; and (ii) the possibility of a “modern” strain disappearing during the first years if there is initially only one infectious individual is extremely high when using discrete solving.

It should also be noted that, in this model, we cannot ascertain the impact of these infections on demographics as a logistic growth has been established. As such, this model has been designed simply to evaluate the competition between both MtbC lineages. Indeed, the predominant “modern” lineages had a marked impact in Paleolithic communities, leading to their eradication, and resulted in slow growth with significant mortality in Neolithic ones. The “protective” effect of populations with stabilized “ancient” epidemy, which stopped the progression of “modern” lineages, is noteworthy and can explain their persistence until modern times despite appearing to have lower fitness.

## Discussion

Our research supports the notion of a significant increase in population growth before the Neolithic period. This comes from dating of the “modern” MtbC lineages to around 43,000 BCE. Indeed, according to our model, the effect of these strains on humanity was brutal. The entrance of an infectious person with a “modern” strain into a typical Paleolithic human group of hunter-gatherers, i.e. groups of 50 persons with a stable “non-growth” status, resulting in their eradication in around 100 years. However, such groups were not isolated and maintained contacts with other groups for mutual help to hunt large animals, exchange information about new territories to explore, or even to interbreed^10^. This means that the sustainability of the group was not simply dependent on the growth in birth rate and also that the infection spread as a result of contact between tribes, thus resulting in the persistence of MtbC and the subsequent disappearance of these groups. A “Neolithic-like” growth by 43,000 BCE similar to that proposed by Miller *et al*.^23^ allows us to understand how “modern” lineages and modern humans were able to persist until modern times.

It should be noted that our results support the proposal that the entrance of “modern” lineages was attenuated by the presence of “ancient” ones. Indeed, the appearance of “ancient” lineages was also a challenge for Paleolithic societies by 73,000 BCE in the context of the most populated continent (Africa) and the different migrations towards the Levant^9^. In order to sustain these societies, we considered a growth rate that could double the population in 100 years in the case of being free from TB, in other words a growth of around 1%/year, which is closer to the Contemporary history growth. This growth is not supported by any previous studies, with reported values not exceeding 0.003%/year^13,52^, unless we include the impact of TB itself on natural mortality. This impact should lead to further research in this field given that a majority of humans were living with TB in the Paleolithic. In fact, after analysing the data, we should maybe have changed change the term “Neolithic” for “Neolithic-like” conditions, although we decided not to do so for the sake of clarity.

One of our key innovations has been to consider the birth rate to be the main source of population growth. This variable is missing in in previous models^28–30^, which simplify it by assuming a logistic growth up to a certain level in order to achieve stabilization of the epidemics. By including this variable, we wanted to understand the impact of TB on demographics, and we have been able to address the question of the lower TB incidence in females. This opens the way to ascertaining the biological mechanisms that make females less susceptible to both TB and other severe epidemics and famines, as has been noted recently^53^. It also opens up eco immunological concepts from the general trade-off between immunity and fertility based on Bateman’s principle of immunity or the “immunocompetence handicap hypothesis”^54,55^ towards a sex-specific investment in defense, depending on the nature of the infecting parasite^56,57^.

That is why we have refined our model assuming a female resistance and tolerance to progression of the infection (s) and to the disease itself (g), respectively, in order to reflect a different incidence between males and females. Indeed, the concept of a balanced response to avoid damage to the host, thus obtaining a better fitness, has been included in the study of human infectious diseases by Casadevall and Pirofski^58^, who clearly showed that an exaggerated response against MtbC is one of the mechanisms for developing active TB. In fact, it is the main factor. Immunosuppression, which can be exemplified by HIV infection as the most extreme and widely distributed example, causes no more than 10% of TB cases worldwide^3^. This knowledge, which has recently been applied to the TB field, has led to a new prophylactic and therapeutic era focused on “host-derived therapies”^59^.

Our data suggest that female resistance predominates over tolerance. This is supported by the data obtained in the Neolithic scenario, which show that only an increase in resistance can explain the reduced incidence in females. The explanation for this resistance could lie in the enhanced level of Tregs generated by oestrogens. As demonstrated in a non-human primate experimental model, latently infected animals with increased levels of Tregs exhibit less progress to active TB^60^. The mechanism is based on the ability of Tregs to reduce local inflammatory responses, especially neutrophil infiltration, which fuels the growth of Mtb^61^. Interestingly, the Tregs-induced tolerance mechanism may be responsible for reducing the bacillary load, as explained in detail previously^62^, thus generating resistance^35^. In this case, for simplicity, we have allocated this property to the ability to develop TB disease, but with a lower mortality. The problem is that the development of TB in females may cause a loss of global human fitness by reducing the reproductive capacity. TB in women generates more extrapulmonary forms than in males, thus resulting in a decrease in fertility of up to 40%. It also increases the perinatal mortality six fold and is the cause of 6–10% of all maternal deaths^63–65^. For simplicity, these data have not been included in our model, which nevertheless supports the concept that a resistance mechanism to avoid the development of TB in women is more likely to explain the co-evolution of humans and MtbC, especially when taking into account the extraordinary consequences on demographics.

One of the main factors affecting the birth rate in the Paleolithic identified by experts is the late weaning of children and the resulting contraceptive effect of breastfeeding^26^. It is still unclear, however, why weaning was brought forward in the agricultural period, thus increasing the birth rate. Although modesty is currently the only explanation, one can speculate the creation of a matriarchal society that can exert demographic control. Indeed, the concept of self-sustainability also appears to be present^10^. As such, we can speculate that TB epidemics stimulated this increase in birth rate, especially after the appearance of “modern” lineages, in a way that selected the communities based on it, thus allowing those that adopted early weaning to persist. Communities with a lower birth rate disappeared.

Our work also has incorporated other assumptions regarding the different virulence mechanisms between “ancient” and “modern” MtbC lineages in the knowledge that this is a question open to discussion. What is clear is the evidence for the higher dissemination ability of “modern” lineages compared with “ancient” ones^21^. The fact that the latter are restricted to specific geographical regions also points to some sort of genetic susceptibility linked to race/ethnic groups, as proposed by several authors^7,17,66^. Our model has not considered this aspect. However, we have established a better ability to infect contacts (e) in “modern” lineages that is twice as high as in “ancient” ones, in the range previously established by Styblo^67^. We have also incorporated a higher fast progression (f) for “ancient” lineages based on their higher pro-inflammatory properties^16^, assuming the limitation of the range of strains used. In this regard, we have hypothesized that “modern” lineages lost the pro-inflammatory ability of “ancient” lineages by decreasing their bacillary size^19^, thus favouring the induction of smaller aerosols and acquiring a better fitness in terms of ability to disseminate.

The uncertainty analysis clearly shows that fast progression (f) is the most important factor as regards increasing the incidence but is also responsible for accelerating clearance. In this regard, a high transmission capacity (e) has a more balanced role by promoting the incidence but having less influence on clearance of the infection in the community. Thus, we can easily explain the success of “modern” lineages even though our results give a predominant role to the reactivation factor, which is higher for “ancient” lineages. Reactivation is an important factor for increasing the incidence while at the same time preventing clearance of the infection. That is why we consider that the balance of both factors (f and a) is very important as regards allowing both the persistence of MtbC and humankind. These findings complement previous hypotheses concerning the relevance of endogenous reactivation when it comes to understanding the persistence of MtbC^27,28^, but refines them by considering reliable experimental data and taking into consideration that, generally speaking, the MtbC in the Paleolithic was quite similar to that found nowadays. This is consistent with the paradigmatic genomic stability found in MtbC^68^. Our analysis also reveals the importance of having a good immunity, thus having the lowest possible value of “i”. In this model, we have used a conservative proxy of the value obtained from the observations reported by Heimbock^69^, which have recently been emphasized by Bloom^70^, concerning the protection triggered by natural MtbC infection against developing active TB (about 97%), and the need to obtain a prophylactic vaccine that can increase this protection while avoiding the risk of fast progression (f) and endogenous reactivation (a).

It is interesting to note the significant effort invested to build a coinfection model applicable to both lineages and to validate the ability of “modern” lineages to replace “ancient” ones in conditions of coinfection. In fact, this can be characterized as a superinfection model as a more virulent strain infects a previously infected host. The disadvantage of more virulent strains is that they kill the host faster, thus causing local extinction of the hosts^71^. The paradox of our model is that we are faced with a lineage that is better able to progress to disease, and thus be transmissible (the “ancient”), and another one with an ability to cause disease but with a greater ability to disseminate (the “modern”). To the best of our knowledge, this is the first attempt to do so. Even when applying a logistic limit to population growth, we have been unable to discern the demographic impact of coinfection, although the model corroborates the higher dissemination of “modern” lineages as a winning strategy while illustrating the protective role of communities previously infected with “ancient” ones. In this regard, the induction of immunity, and the reduction in the susceptible population, explains this phenomenon. It also explains how “ancient” lineages have been able to persist when faced with such competition, becoming limited geographically^21^. In this regard, the incidence of “ancient” covers the territories implicated in the second “out of Africa” migration, from West Africa towards India and Australia^9^.

In summary, our model shows the marked impact of TB on human history, from the Paleolithic, a fact that should be revisited and included in future studies to interpret this human history. Our model agrees with the more recent data showing a demographic explosion prior to the Neolithic revolution. We also consider factors such as the higher vulnerability of recovered patients which, especially under current conditions, still represent 40–50% of undiagnosed TB cases in Asia and Africa. Finally, we highlight the importance of female resistance to understand the lower incidence in women, a fact that should be studied in order to discern its biological basis.

## Methodology

### Basic TB natural history compartmental model

We have designed a compartmental mathematical model based on five differential equations to describe the dynamics of the evolution of MtbC infection in the population, based on previous models^28–30,51^ (Supplementary Figure 1 and Table 1). We have classified the whole population (N) into compartments following the classical SEIR [susceptible (S), exposed/infected (E), infectious (I) and recovered (R)] approach, where $N=S+E+I+R$. The evolution of these five variables is described by Eqs. (1–5):

1 $$\frac{dS}{dt}=\pi +\delta E-\mu S-\beta IS$$

2 $$\frac{dE}{dt}=(1-f)\,\beta SI\,-(\mu +\delta +i(a+rI))E$$

3 $$\frac{dI}{dt}=f\beta SI+i(a+rI)E+wi(a+rI)R-(\mu +{\mu }_{TB}+c)I$$

4 $$\frac{dR}{dt}=cI-(\mu +wi(a+rI))R$$

5 $$\frac{dN}{dt}=\frac{dS}{dt}+\frac{dE}{dt}+\frac{dI}{dt}+\frac{dR}{dt}$$

Infected aerosols are released by infectious subjects (I) and can infect susceptible individuals (S) or reinfect already infected ones (E and R), with an annual risk of infection (*β*) that depends on the number of new infections (*e*) caused by a particular case (I). This is related to the growth ratio of the population, and thus the birth rate (*π*) and natural mortality (*μ*). Thus, the annual risk of infection is described by the relationship $\beta =e\cdot \pi /\mu$^30^.

After infection or reinfection, subjects can develop the disease during the first year according to the probability *f*, known as fast progression. This probability has been taken from the most recent studies concerning progression from infection to disease^45,72^. The most detailed of these studies^45^ gives a value of 8.25% for the whole population. Furthermore, once an infected (E) or recovered status (R) has been achieved, there is a chance of reactivation during the following four years, which according to these authors represents 1.5% of the fast progression (*f*) and is termed the reactivation factor (*a*). In light of this, we have defined the risk of disease caused by reinfection (*r*) as $r=f\beta +a(1-f)\beta$. This value is substantially decreased by the immunity (*i*) generated by infection. Considering the studies of Heimbeck^69^, those infected people that do not develop the disease have a protection against the onset of disease of at least 90%, thus resulting in a protective ratio ($i$) of 0.1.

Infected people (E) can both drain the bacillary load and reduce the immunity (*δ*), depending on the dynamic nature of the infection^41^ during the period of around 10 years established in BCG immunity studies^40^. This would lead to a drainage probability of about 0.1 annually, although this is reduced by the possibility of endogenous or exogenous reactivation of the infection, defined as $a$ and $r$, respectively. Thus, bacillary drainage is defined as: $\delta$ = 0.1 – *a* – *r*.

According to Tiemerman *et al*.^47^, TB patients can cure naturally in three years, thus giving an annual curation rate (*c*) of 0.33. They also have a global chance of dying of 45% during these three years, therefore the annual mortality caused by TB (*μ*_TB_) is 0.15. Furthermore, Uys *et al*.^31^ have determined that recovered subjects have a sevenfold higher chance of developing disease, thus we have included this factor (*w*) in our model. The birth rate (*π*) is determined from the mean number of births per fertile woman/year (*λ*).

In light of the previous findings of Bold *et al*. and Portevin *et al*.^16,19^, we assumed that, given their ability to induce a higher proinflammatory response to macrophages, “ancient” lineages were able to increase the probability of fast progression (*f*) and reactivation (*a*) more than their “modern” counterparts. The percentage increase resulting from the higher inflammation was established as 20% as this is the relative volume of the upper lobes, with this property being determinant for the onset of disease^18^. We consider that the qualitative advantage of “modern” lineages arises due to the reduction of the size of the bacilli^19^. As such, “modern” lineages replace the “ancient” pro-inflammatory advantage by developing more infective aerosols, thus substantially increasing the number of infections caused by a patient (*e*). Given the findings of Styblo^67^, we have situated these values as the average in the case of “ancient” lineages and the upper limit for the “modern” ones, in other words a value of 10 and 20, respectively.

Our model considers the concepts of resistance to infection, which limits the progression to disease (i.e. reducing the bacillary load), and tolerance to disease, which limits the mortality caused by TB ($\mu$_TB_). This is because tolerance reduces the damage caused by MtbC infection and increases the fitness of the host^58^. In order to quantify these differences in general mortality between the Paleolithic and Neolithic periods, we have considered the difference in life expectancy, estimated as 33 and 26.5 years, respectively^73,74^. This 25% difference has been taken into account to determine the change in resistance, thus affecting the fast progression (*f*) and reactivation factor (*a*), and changes in tolerance, thus impacting TB-related mortality (*μ*_TB_).

The reader can find a figure with the model’s flow chart and a table with the parameters’ values in the article (Fig. 1A and Table 1).

### TBOREX model

In order to study the observed sex-related variations in incidence, we have refined the basic model by building the TBOREX (TB, Origen and Sex) one, which take into consideration male (M) and female (F) subpopulations in each compartment [susceptible (S), exposed/infected (E), infectious (I) and recovered (R)]. This results in the following differential Eqs. (6–15),

6 $$\frac{d{S}_{M}}{dt}=\pi +\delta {E}_{M}-\mu {S}_{M}-\beta ({I}_{M}+{I}_{F}){S}_{M}$$

7 $$\frac{d{E}_{M}}{dt}=(1-fs)\beta ({I}_{M}+{I}_{F}){S}_{M}-(\mu +\delta +is(a+r({I}_{M}+{I}_{F}))){E}_{M}$$

8 $$\begin{array}{c}\frac{d{I}_{M}}{dt}=sf\beta ({I}_{M}+{I}_{F}){S}_{M}+is(a+r({I}_{M}+{I}_{F})){E}_{M}+wis(a+r({I}_{M}+{I}_{F})){R}_{M}\\ \,\,\,-\,(\mu +g{\mu }_{TB}+c){I}_{M}\end{array}$$

9 $$\frac{d{R}_{M}}{dt}=c{I}_{M}-(\mu +wis(a+r({I}_{M}+{I}_{F}))){R}_{M}$$

10 $$\frac{d{N}_{M}}{dt}=\frac{d{S}_{M}}{dt}+\frac{d{E}_{M}}{dt}+\frac{d{I}_{M}}{dt}+\frac{d{R}_{M}}{dt}$$

11 $$\frac{d{S}_{F}}{dt}=\pi +\delta {E}_{F}-\mu {S}_{F}-\beta ({I}_{M}+{I}_{F}){S}_{F}$$

12 $$\frac{d{E}_{F}}{dt}=(1-\frac{1}{s}f)\beta ({I}_{M}+{I}_{F}){S}_{F}-(\mu +\delta +i\frac{1}{s}(a+r({I}_{M}+{I}_{F}))){E}_{F}$$

13 $$\begin{array}{c}\frac{d{I}_{F}}{dt}=\frac{1}{s}f\beta ({I}_{M}+{I}_{F}){S}_{F}+i\frac{1}{s}(a+r({I}_{M}+{I}_{F})){E}_{F}\\ \,\,\,\,+wi\frac{1}{s}(a+r({I}_{M}+{I}_{F})){R}_{F}-(\mu +\frac{1}{g}{\mu }_{TB}+c){I}_{F}\end{array}$$

14 $$\frac{d{R}_{F}}{dt}=c{I}_{F}-(\mu +wi\frac{1}{s}(a+r({I}_{M}+{I}_{F}))){R}_{F}$$

15 $$\frac{d{N}_{F}}{dt}=\frac{d{S}_{F}}{dt}+\frac{d{E}_{F}}{dt}+\frac{d{I}_{F}}{dt}+\frac{d{R}_{F}}{dt}$$

The total population values in each compartment can be evaluated simply by adding both subpopulations (e.g., $N={N}_{M}+{N}_{F}$).

There is a wide consensus regarding the stability of the population density in the Middle Paleolithic. Thus, it appears that the growth rate was around 0.003%, which significantly increased up to the 0.1% in the Neolithic^13,26,52^. We have adjusted this rate for the expected predominant lineage in each period, namely the “ancient” one for the Paleolithic and the “modern” for the Neolithic, to determine the birth rate ($\lambda$). This gave a value of $\lambda$ = 0.0778, which means an average of 2.8 children/female in the Paleolithic period. For the Neolithic, we considered a value of $\lambda$ = 0.128, thus meaning an average of 4.7 children/female. In both cases we considered that 50% of females are fertile every year, with a fertility period of 18 years starting at the age of 15. These fertility values give a much higher population growth than that determined by different experts in this field^13,14,26,52^. The birth rate in each susceptible compartment (susceptible male and susceptible female) can be represented as $\pi =0.5\cdot \lambda \cdot {N}_{F}$, where ${N}_{F}$ represents the total number of females. In order to maintain the actual female/male incidence proportion at the accepted value of 60/40, we adjusted the progression to disease (resistance) by a factor (s) that increases or reduces this in men and women, respectively, in a proportion of 55/45. We also added this proportion to the mortality caused by TB (*μ*_TB_) in order to reproduce tolerance to disease (g).

The reader can find a figure with the model’s flow chart and a table with the parameters’ values in the article (Fig. 1B and Table 1).

### Assessment of uncertainty and sensitivity in the system

An uncertainty and sensitivity analysis were performed for the TBOREX model as described in^75^ using a sampling-based method. Thus, 1000 different parameter sets were used to explore the space using a Latin Hipercube Sampling (LHS) technique. Parameters were explored between the values shown in Table 2. At each time step, the Partial Rank Correlation Coefficient (PRCC) was computed for each of the parameters and susceptible, exposed, infected, recovered and total populations, as well as the annual incidence and death rate. The final PRCC between input parameters and TB clearance was also computed, using the discrete resolution. This methodology allowed us to see how each output was affected upon increasing (or decreasing) a specific parameter (linearly discounting the effects of the uncertainty on the rest of the parameters). Thus, PRCC can be used to determine which parameters to target to achieve specific goals.

### Coinfection model

The coinfection model takes into consideration the coexistence of “ancient” (*a*) and “modern” (*m*) lineages in a certain community. The variables of the model are the susceptible individuals (S), the exposed (E), the infectious (I) and the recovered (R). ${E}_{a}{S}_{m}$, ${S}_{a}{E}_{m}$ and ${E}_{a}{E}_{m}$ represent persons with an ancient, a modern or both latent infections, respectively. The same nomenclature is applied to all possible combinations between *S*_*a*_, *E*_*a*_, *I*_*a*_ and *R*_*a*_ – i.e., TB compartments related with ancient strain – and ${S}_{m}$, ${E}_{m}$, ${I}_{m}$ and ${R}_{m}$– i.e., TB compartments related with modern strain. For simplicity, in these equations we will use: ${I}_{a}={I}_{a}{S}_{m}+{I}_{a}{E}_{m}+{I}_{a}{R}_{m}$ (i.e., all the compartments with ancient strain TB-sick); ${I}_{m}={S}_{a}{I}_{m}+{E}_{a}{I}_{m}+{R}_{a}{I}_{m}$ (i.e., all the compartments with modern strain TB-sick); ${I}_{am}={I}_{a}{I}_{m}$ (i.e., the compartment with TB-sick with the two strains). The evolution of all variables is described by Eqs. (16–31):

16 $$\begin{array}{c}\frac{d}{dt}{S}_{a}{S}_{m}=\Pi -{\beta }_{a}\cdot {S}_{a}{S}_{m}\cdot {I}_{a}-{\beta }_{m}\cdot {S}_{a}{S}_{m}\cdot {I}_{m}+{\delta }_{m}\cdot {S}_{a}{E}_{m}+{\delta }_{a}\cdot {E}_{a}{S}_{m}-\mu \\ \,\,\,\,\cdot {S}_{a}{S}_{m}\end{array}$$

17 $$\begin{array}{c}\frac{d}{dt}{E}_{a}{S}_{m}={\delta }_{m}\cdot {E}_{a}{E}_{m}-i({a}_{a}+{r}_{a}{I}_{a})\cdot {E}_{a}{S}_{m}+(1-{f}_{a}){\beta }_{a}\cdot {S}_{a}{S}_{m}\cdot {I}_{a}-{\beta }_{m}\\ \,\,\,\,\cdot {E}_{a}{S}_{m}\cdot {I}_{m}-{\delta }_{a}\cdot {E}_{a}{S}_{m}+{\delta }_{m}\cdot {E}_{a}{E}_{m}-\mu \cdot {E}_{a}{S}_{m}\end{array}$$

18 $$\begin{array}{c}\frac{d}{dt}{I}_{a}{S}_{m}={\delta }_{m}\cdot {I}_{a}{E}_{m}-c\cdot {I}_{a}{S}_{m}+wi({a}_{a}+{r}_{a}{I}_{a})\cdot {R}_{a}{S}_{m}+i({a}_{a}+{r}_{a}{I}_{a})\cdot {E}_{a}{S}_{m}\\ \,\,\,\,+{f}_{a}{\beta }_{a}\cdot {S}_{a}{S}_{m}\cdot {I}_{a}-{\beta }_{m}\cdot {S}_{a}{S}_{m}\cdot {I}_{m}-(\mu +{\mu }_{TB})\cdot {I}_{a}{S}_{m}\end{array}$$

19 $$\begin{array}{c}\frac{d}{dt}{R}_{a}{S}_{m}={\delta }_{m}\cdot {R}_{a}{E}_{m}-wi({a}_{a}+{r}_{a}{I}_{a})\cdot {R}_{a}{S}_{m}+c\cdot {I}_{a}{S}_{m}-{\beta }_{m}\cdot {S}_{a}{S}_{m}\cdot {I}_{m}-\mu \\ \,\,\,\,\,\cdot {R}_{a}{S}_{m}\end{array}$$

20 $$\begin{array}{c}\frac{d}{dt}{S}_{a}{E}_{m}={\delta }_{a}\cdot {E}_{a}{E}_{m}-{\delta }_{m}\cdot {S}_{a}{E}_{m}-i({a}_{m}+{r}_{m}{I}_{m})\cdot {S}_{a}{E}_{m}-{\beta }_{a}\cdot {S}_{a}{S}_{m}\cdot {I}_{a}\\ \,\,\,\,+(1-{f}_{m}){\beta }_{m}\cdot {S}_{a}{S}_{m}\cdot {I}_{m}-\mu \cdot {S}_{a}{E}_{m}\end{array}$$

21 $$\begin{array}{c}\frac{d}{dt}{E}_{a}{E}_{m}=-{\delta }_{a}\cdot {E}_{a}{E}_{m}-{\delta }_{m}\cdot {E}_{a}{E}_{m}-i({a}_{a}+{r}_{a}{I}_{a})\cdot {E}_{a}{E}_{m}-i({a}_{m}+{r}_{m}{I}_{m})\\ \,\,\,\,\cdot {E}_{a}{E}_{m}+(1-{f}_{a}){\beta }_{a}\cdot {S}_{a}{E}_{m}\cdot {I}_{a}+(1-{f}_{m}){\beta }_{m}\cdot {E}_{a}{S}_{m}\cdot {I}_{m}-\mu \,\cdot {E}_{a}{E}_{m}\end{array}$$

22 $$\begin{array}{c}\frac{d}{dt}{I}_{a}{E}_{m}=-\,{\delta }_{m}\cdot {I}_{a}{E}_{m}+wi({a}_{a}+{r}_{a}{I}_{a})\cdot {R}_{a}{E}_{m}-c\cdot {I}_{a}{E}_{m}+i({a}_{a}+{r}_{a}{I}_{a})\\ \,\,\,\,\cdot {E}_{a}{E}_{m}-i({a}_{m}+{r}_{m}{I}_{m})\cdot {I}_{a}{E}_{m}+{f}_{a}{\beta }_{a}\cdot {S}_{a}{E}_{m}\cdot {I}_{a}+(1-{f}_{m}){\beta }_{m}\\ \,\,\,\,\cdot {I}_{a}{S}_{m}\cdot {I}_{m}-(\mu +{\mu }_{TB})\cdot {I}_{a}{E}_{m}\end{array}$$

23 $$\begin{array}{c}\frac{d}{dt}{R}_{a}{E}_{m}=-\,{\delta }_{m}\cdot {R}_{a}{E}_{m}-wi({a}_{a}+{r}_{a}{I}_{a})\cdot {R}_{a}{E}_{m}+c\cdot {I}_{a}{E}_{m}-i({a}_{m}+{r}_{m}{I}_{m})\\ \,\,\,\,\cdot {R}_{a}{E}_{m}+(1-{f}_{m}){\beta }_{m}\cdot {R}_{a}{S}_{m}\cdot {I}_{m}-\mu \cdot {R}_{a}{E}_{m}\end{array}$$

24 $$\begin{array}{c}\frac{d}{dt}{S}_{a}{I}_{m}={\delta }_{a}\cdot {E}_{a}{I}_{m}+wi({a}_{m}+{r}_{m}{I}_{m})\cdot {S}_{a}{R}_{m}-c\cdot {S}_{a}{I}_{m}+i({a}_{m}+{r}_{m}{I}_{m})\\ \,\,\,\,\cdot {S}_{a}{E}_{m}-{\beta }_{a}\cdot {S}_{a}{S}_{m}\cdot {I}_{a}+{f}_{m}{\beta }_{m}\cdot {S}_{a}{S}_{m}\cdot {I}_{m}-(\mu +{\mu }_{TB})\cdot {S}_{a}{I}_{m}\end{array}$$

25 $$\begin{array}{c}\frac{d}{dt}{E}_{a}{I}_{m}=-\,{\delta }_{a}\cdot {E}_{a}{I}_{m}+wi({a}_{m}+{r}_{m}{I}_{m})\cdot {E}_{a}{R}_{m}-c\cdot {E}_{a}{I}_{m}-i({a}_{a}+{r}_{a}{I}_{a})\\ \,\,\,\,\cdot {E}_{a}{I}_{m}+i({a}_{m}+{r}_{m}{I}_{m})\cdot {E}_{a}{E}_{m}+(1-{f}_{a}){\beta }_{a}\cdot {S}_{a}{I}_{m}\cdot {I}_{a}+{f}_{m}{\beta }_{m}\\ \,\,\,\,\cdot {E}_{a}{S}_{m}\cdot {I}_{m}-(\mu +{\mu }_{TB})\cdot {E}_{a}{I}_{m}\end{array}$$

26 $$\begin{array}{c}\frac{d}{dt}{I}_{a}{I}_{m}=wi({a}_{m}+{r}_{m}{I}_{m})\cdot {I}_{a}{R}_{m}+wi({a}_{a}+{r}_{a}{I}_{a})\cdot {R}_{a}{I}_{m}-2c\cdot {I}_{a}{I}_{m}\\ \,\,\,\,+i({a}_{a}+{r}_{a}{I}_{a})\cdot {E}_{a}{I}_{m}+i({a}_{m}+{r}_{m}{I}_{m})\cdot {I}_{a}{E}_{m}+{f}_{a}{\beta }_{a}\cdot {S}_{a}{I}_{m}\cdot {I}_{a}\\ \,\,\,\,+{f}_{m}{\beta }_{m}\cdot {I}_{a}{S}_{m}\cdot {I}_{m}-(\mu +2{\mu }_{TB})\cdot {I}_{a}{I}_{m}\end{array}$$

27 $$\begin{array}{c}\frac{d}{dt}{R}_{a}{I}_{m}=wi({a}_{m}+{r}_{m}{I}_{m})\cdot {R}_{a}{R}_{m}-wi({a}_{a}+{r}_{a}{I}_{a})\cdot {R}_{a}{I}_{m}+c\cdot {I}_{a}{I}_{m}-c\cdot {R}_{a}{I}_{m}\\ \,+i({a}_{m}+{r}_{m}{I}_{m})\cdot {R}_{a}{E}_{m}+{f}_{m}{\beta }_{m}\cdot {R}_{a}{S}_{m}\cdot {I}_{m}-(\mu +{\mu }_{TB})\cdot {R}_{a}{I}_{m}\end{array}$$

28 $$\frac{d}{dt}{S}_{a}{R}_{m}={\delta }_{a}\cdot {E}_{a}{R}_{m}-wi({a}_{m}+{r}_{m}{I}_{m})\cdot {S}_{a}{R}_{m}+c\cdot {S}_{a}{I}_{m}-{\beta }_{a}\cdot {S}_{a}{S}_{m}\cdot {I}_{a}-\mu \cdot {S}_{a}{R}_{m}$$

29 $$\begin{array}{c}\frac{d}{dt}{E}_{a}{R}_{m}=-{\delta }_{a}\cdot {E}_{a}{R}_{m}-wi({a}_{m}+{r}_{m}{I}_{m})\cdot {E}_{a}{R}_{m}+c\cdot {E}_{a}{I}_{m}-i({a}_{a}+{r}_{a}{I}_{a})\\ \,\cdot {E}_{a}{R}_{m}+(1-{f}_{a}){\beta }_{a}\cdot {S}_{a}{R}_{m}\cdot {I}_{a}-\mu \cdot {E}_{a}{R}_{m}\end{array}$$

30 $$\begin{array}{c}\frac{d}{dt}{I}_{a}{R}_{m}=-wi({a}_{m}+{r}_{m}{I}_{m})\cdot {I}_{a}{R}_{m}+wi({a}_{a}+{r}_{a}{I}_{a})\cdot {R}_{a}{R}_{m}-c\\ \,\cdot {I}_{a}{R}_{m}+c\cdot {I}_{a}{I}_{m}+i({a}_{a}+{r}_{a}{I}_{a})\cdot {E}_{a}{R}_{m}+{f}_{a}{\beta }_{a}\cdot {S}_{a}{R}_{m}\cdot {I}_{a}-(\mu +{\mu }_{TB})\cdot {I}_{a}{R}_{m}\end{array}$$

31 $$\begin{array}{c}\frac{d}{dt}{R}_{a}{R}_{m}=-wi({a}_{m}+{r}_{m}{I}_{m})\cdot {R}_{a}{R}_{m}-wi({a}_{a}+{r}_{a}{I}_{a})\cdot {R}_{a}{R}_{m}+c\cdot {I}_{a}{R}_{m}+c\\ \,\cdot {R}_{a}{I}_{m}-\mu \cdot {R}_{a}{R}_{m}\end{array}$$

where:

$${r}_{a}={f}_{a}{\beta }_{a}+{a}_{a}(1-{f}_{a}){\beta }_{a}$$ $${r}_{m}={f}_{m}{\beta }_{m}+{a}_{m}(1-{f}_{m}){\beta }_{m}$$

$${\delta }_{a}=0.1-{a}_{a}-{r}_{a}$$ $${\delta }_{m}=0.1-{a}_{m}-{r}_{m}$$

$${\beta }_{a}={e}_{a}\mu /\pi $$ $${\beta }_{m}={e}_{m}\mu /\pi $$

The reader can find a figure with the model’s flow chart and a table with the parameters’ values in the article (Fig. 2 and Table 1).

### Continuous and discrete resolution of the models

The two models were numerically integrated with Matlab using the Euler method, with an integration step of 1/36 years. This resulted in curves showing the evolution of each variable, as well as the annual incidence and mortality. The models were also resolved using Matlab’s ode45 package (MathWorks, Natick, Massachusetts, EEUU) to verify the correctness of the manual resolution.

The limited size of some of the communities studied suggests the suitability of exploring a discrete resolution of the models, using natural numbers to describe the variable dynamics. To that end, discrete resolution was implemented using Euler’s integration method but converting each of the flows at each integration step into a natural number using Poisson random distribution. As such, discrete resolution of the models provides a different solution due to the effect of randomness.

## Supplementary information

supporting information.
