# Supplementary material for: Origin of tuberculosis in the Paleolithic predicts unprecedented population growth and female resistance
Source: Sci Rep. 2020 Jan 8;10:42. doi: 10.1038/s41598-019-56769-1 (PMC6949267; doi:10.1038/s41598-019-56769-1)
Supplement: Supplementary file 1 — supporting information. [file 41598_2019_56769_MOESM1_ESM.pdf]

**Supplementary file**

# Origin of tuberculosis in the Paleolithic predicts unprecedented population growth and female resistance.

*Pere-Joan Cardona[1], Martí Català[2], Clara Prats[3].

[1]Unitat de Tuberculosi Experimental, Institut de Recerca Germans Trias i Pujol (IGTP), Universitat Autònoma de Barcelona, CIBERES. Badalona, Catalonia, Spain. [2]Comparative Medicine and Bioimage Centre of Catalonia (CMCiB). Fundació Institut d'Investigació en Ciències de la Salut Germans Trias i Pujol. Badalona, Catalonia, Spain. [3]Escola Superior d'Agricultura de Barcelona, Departament de Física, Universitat Politècnica de Catalunya (UPC)-BarcelonaTech. Castelldefels, Catalonia, Spain.

*Correspondence author.
e-mail: pj.cardona@gmail.com

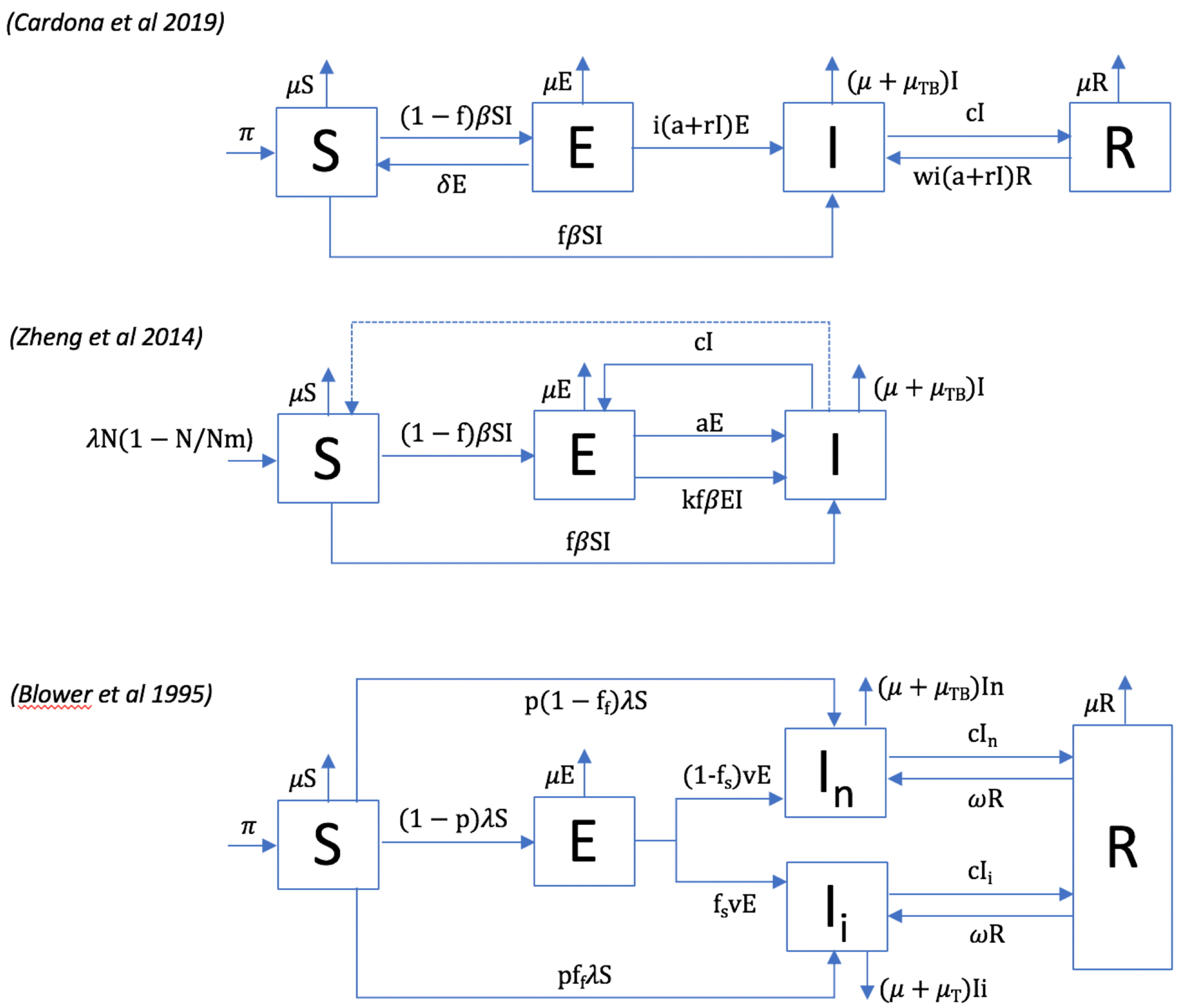


## Supplementary Figure 1

**Comparison of the basic SEIR model with those from bibliography.** Each compartment refers to the set of individuals by disease status: Susceptible (S), Infected (E), Sick (I) and Recovered (R). The model from Blower et al distinguishes between infectious and non-infectious diseased. Arrows represent the flows among compartments. See original references for details on the models assumptions, parameters and equations.

## Supplementary Figure 2

**Evolution of the population in the discrete model TBOREX after transforming values to natural numbers**. Pictures show the projections for 100 years in initial populations of 1000 members where a unique male TB patient is included ($I_o$=1).

Evolution is drawn under Neolithic life conditions, in relation to the MtbC lineage infection, "Ancient" (**A**) or "Modern" (**B**). Continuous lines show the total population (**N**) (**black**), Non-infected susceptible (**S**) (**green**), Infected (**E**) (**blue**), TB patients Infectious (**I**) (**red**) and previous TB patients Recovered (**R**) (**orange**). Dotted lines represents reference values. For the population, we have considered 2 and 10 persons; for the time, 3 and 10 years.

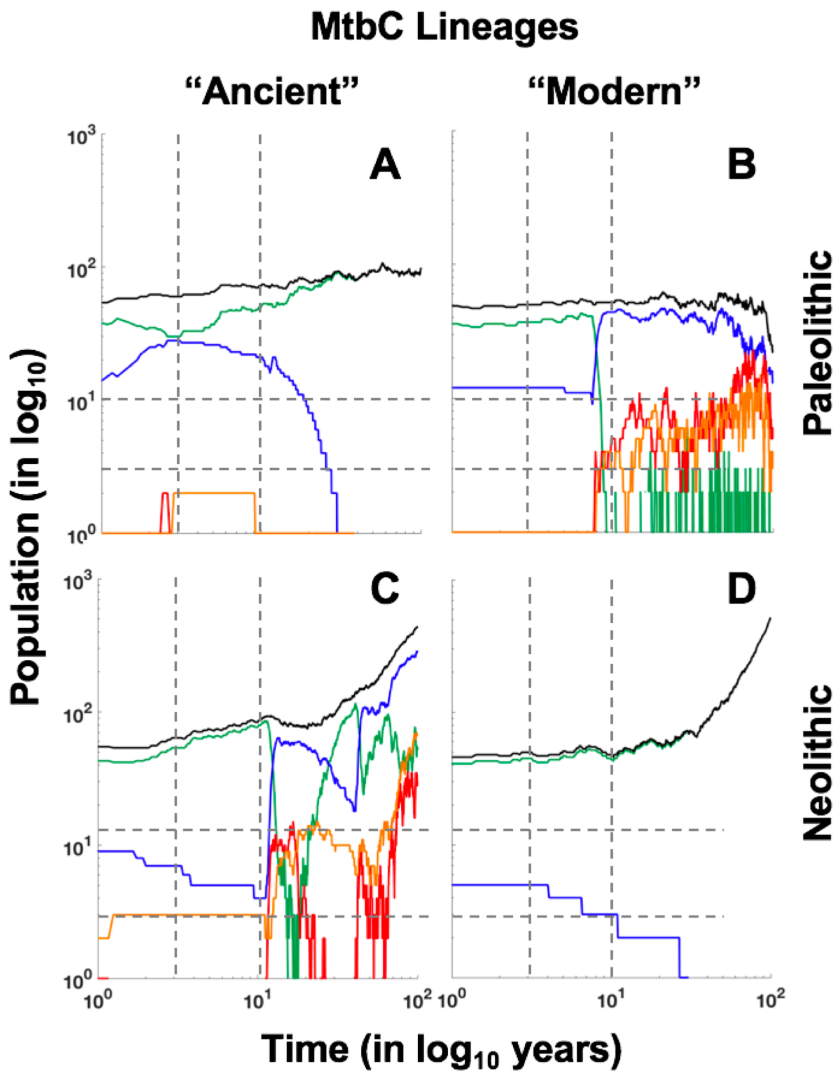

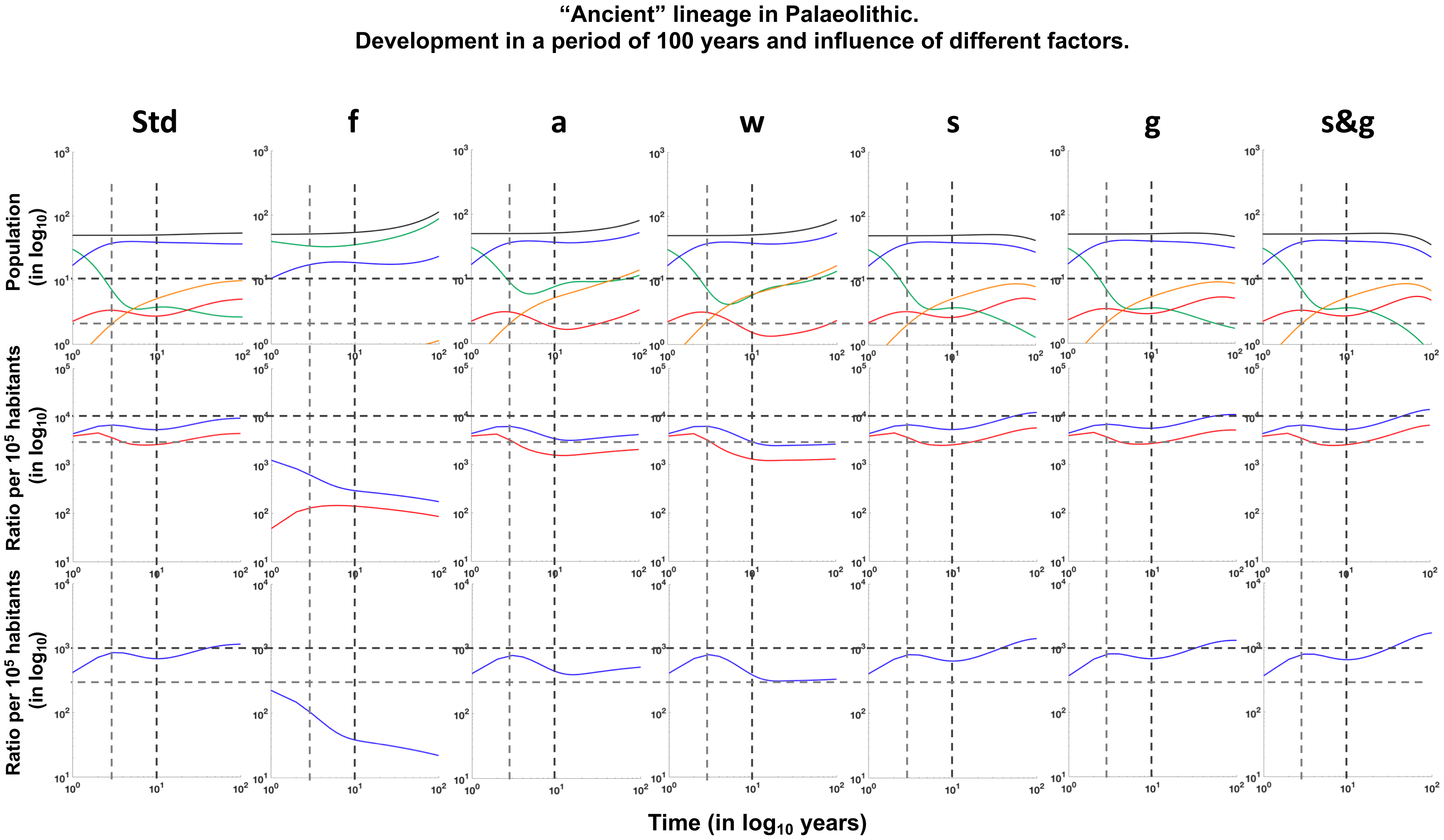


## Supplementary Figure 3

**Influence of different factors on a 100-years evolution of "Ancient" lineage in a Palaeolithic community. Std** stands for the standard simulation with the parameters detailed in the text (Table 1). Other columns represent the inhibition of each of the factors; **f**: without fast progression (f=0); **a**: without reactivation from infection (a=0); **w**: without an increase in progression probability in Recovered (w=1); **s**: without male/female differences on disease progression (s=1); **g**: without male/female differences on disease tolerance (g=1); **s&g**: without male/female differences (s=g=1).

“Modern” lineage in Palaeolithic.
Development in a period of 100 years and influence of different factors.

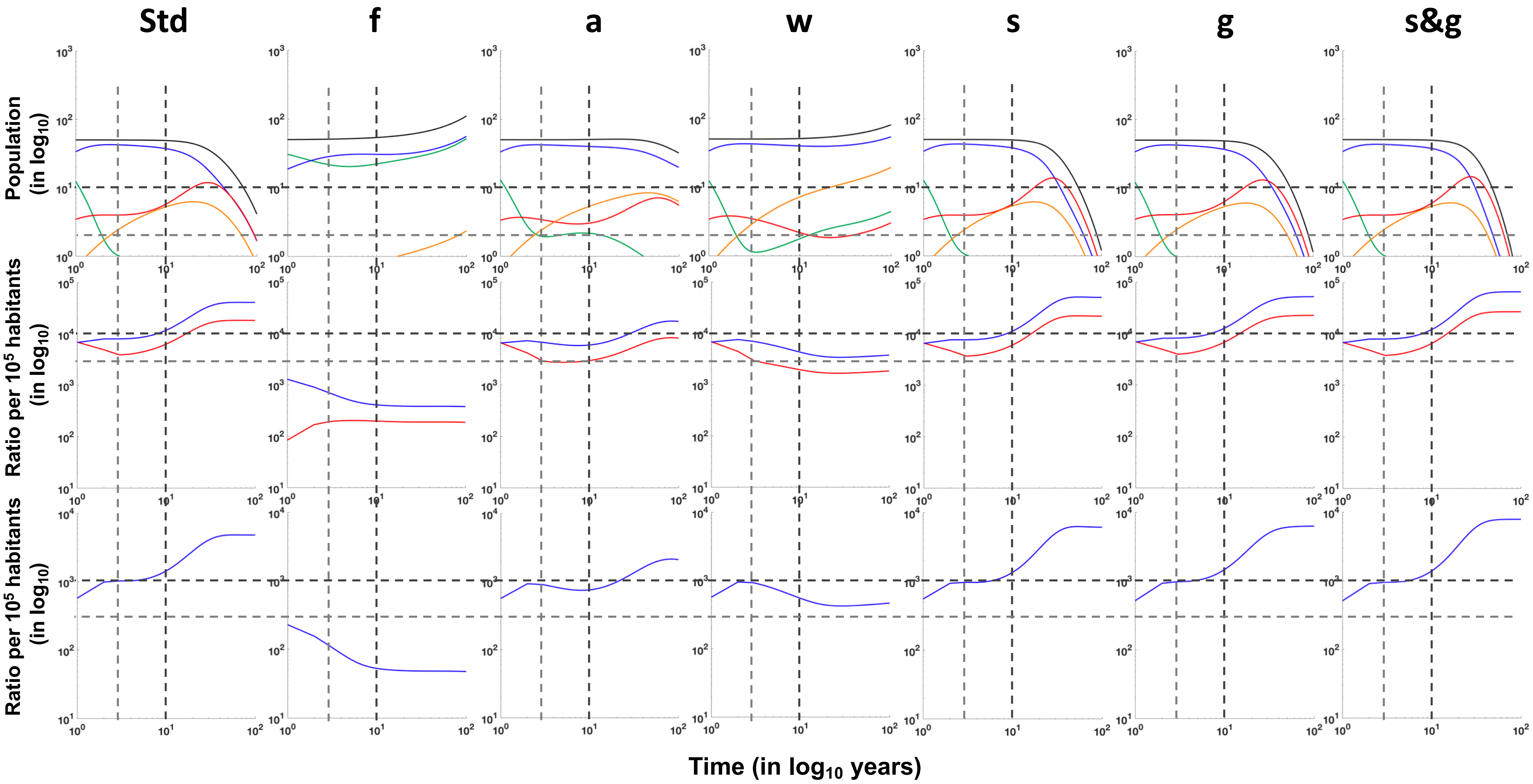


## Supplementary Figure 4

**Influence of different factors on a 100-years evolution of “Modern” lineage in a Palaeolithic community. Std** stands for the standard simulation with the parameters detailed in the text (Table 1). Other columns represent the inhibition of each of the factors; **f**: without fast progression (f=0); **a**: without reactivation from infection (a=0); **w**: without an increase in progression probability in Recovered (w=1); **s**: without male/female differences on disease progression (s=1); **g**: without male/female differences on disease tolerance (g=1); **s&g**: without male/female differences (s=g=1).

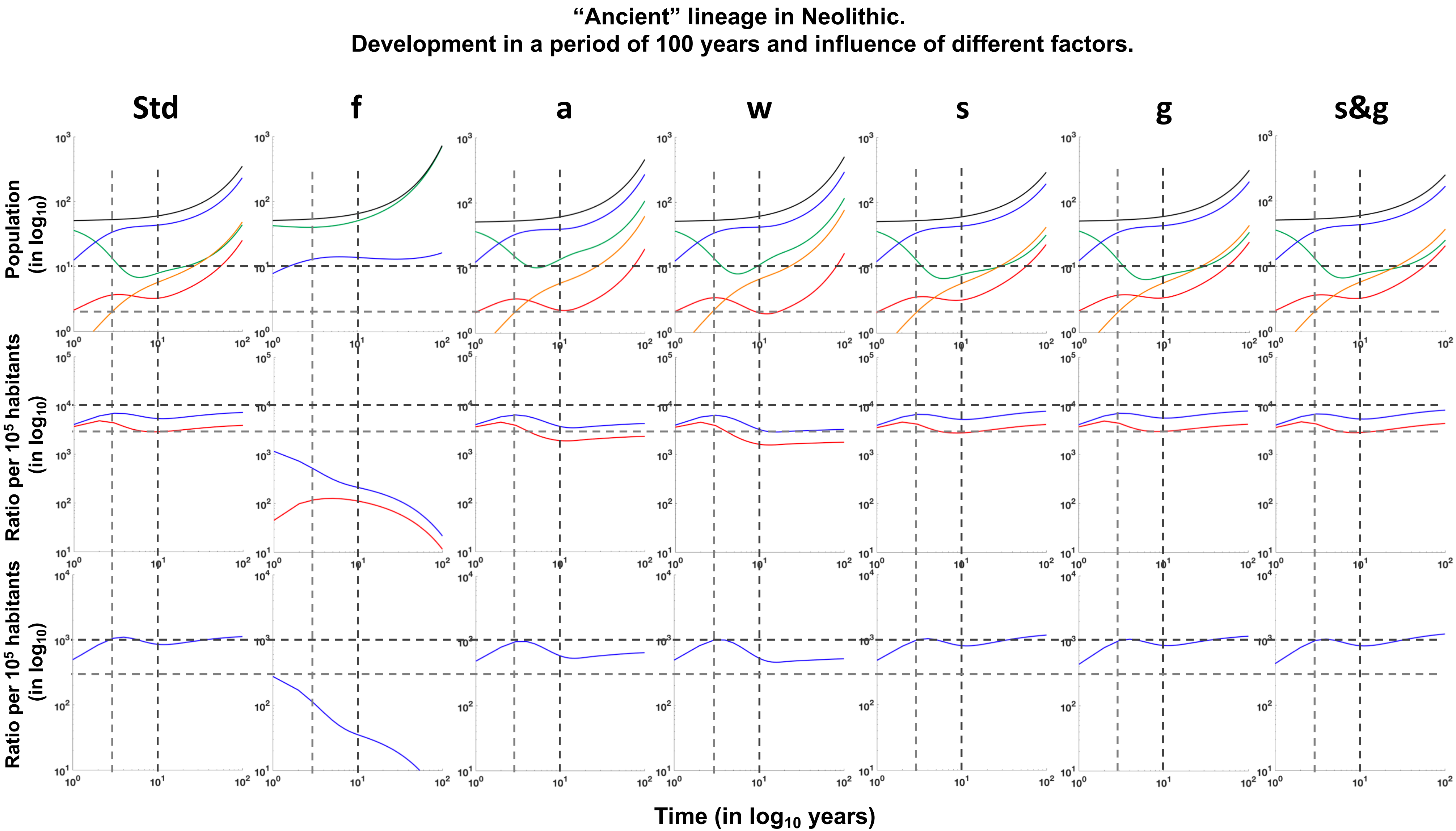


## Supplementary Figure 5

**Influence of different factors on a 100-years evolution of "Ancient" lineage in a Neolithic community. Std** stands for the standard simulation with the parameters detailed in the text (Table 1). Other columns represent the inhibition of each of the factors; **f**: without fast progression (f=0); **a**: without reactivation from infection (a=0); **w**: without an increase in progression probability in Recovered (w=1); **s**: without male/female differences on disease progression (s=1); **g**: without male/female differences on disease tolerance (g=1); **s&g**: without male/female differences (s=g=1).

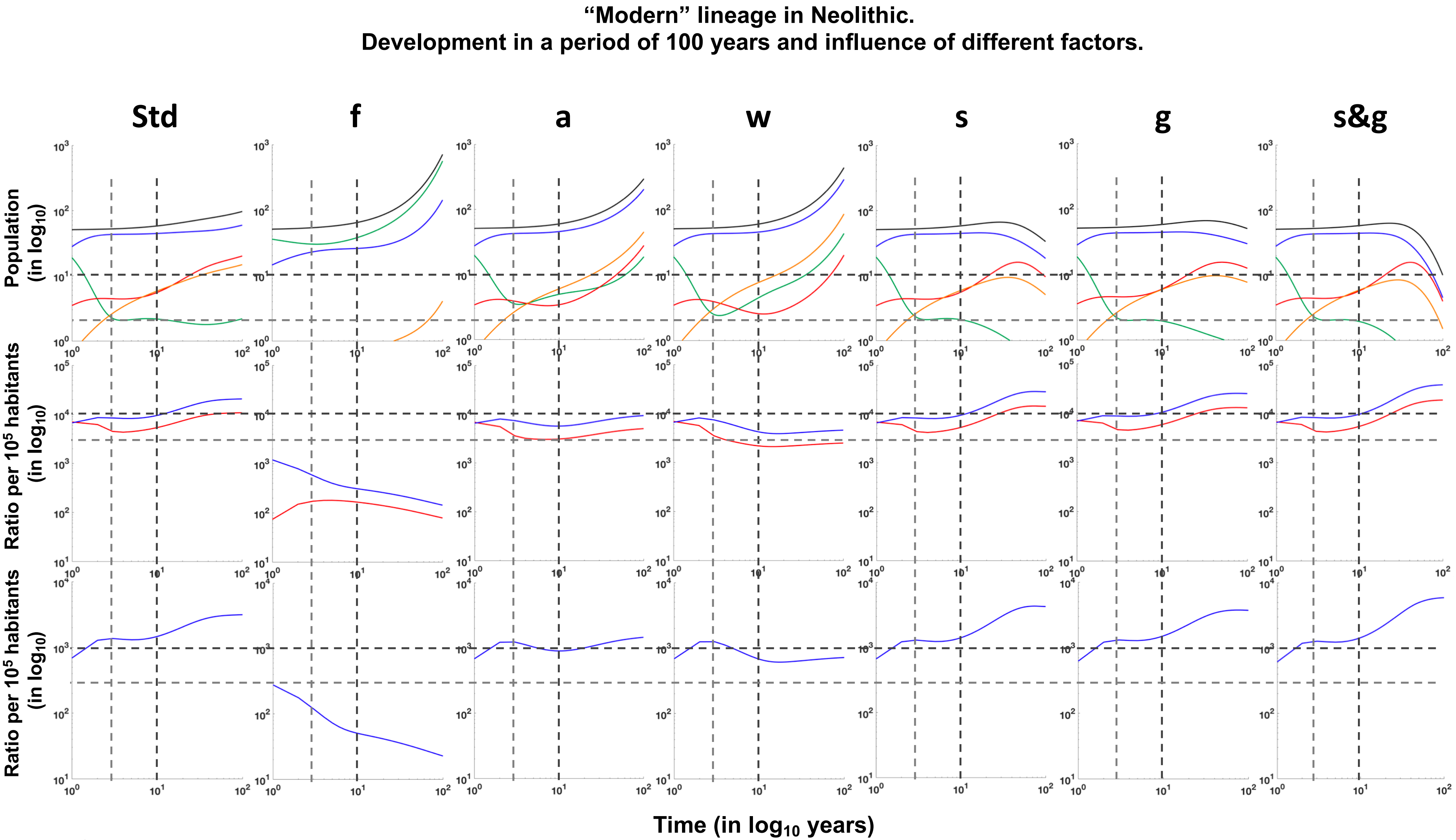


## Supplementary Figure 6

**Influence of different factors on a 100-years evolution of "Modern" lineage in a Neolithic community. Std** stands for the standard simulation with the parameters detailed in the text (Table 1). Other columns represent the inhibition of each of the factors; **f**: without fast progression (f=0); **a**: without reactivation from infection (a=0); **w**: without an increase in progression probability in Recovered (w=1); **s**: without male/female differences on disease progression (s=1); **g**: without male/female differences on disease tolerance (g=1); **s&g**: without male/female differences (s=g=1).

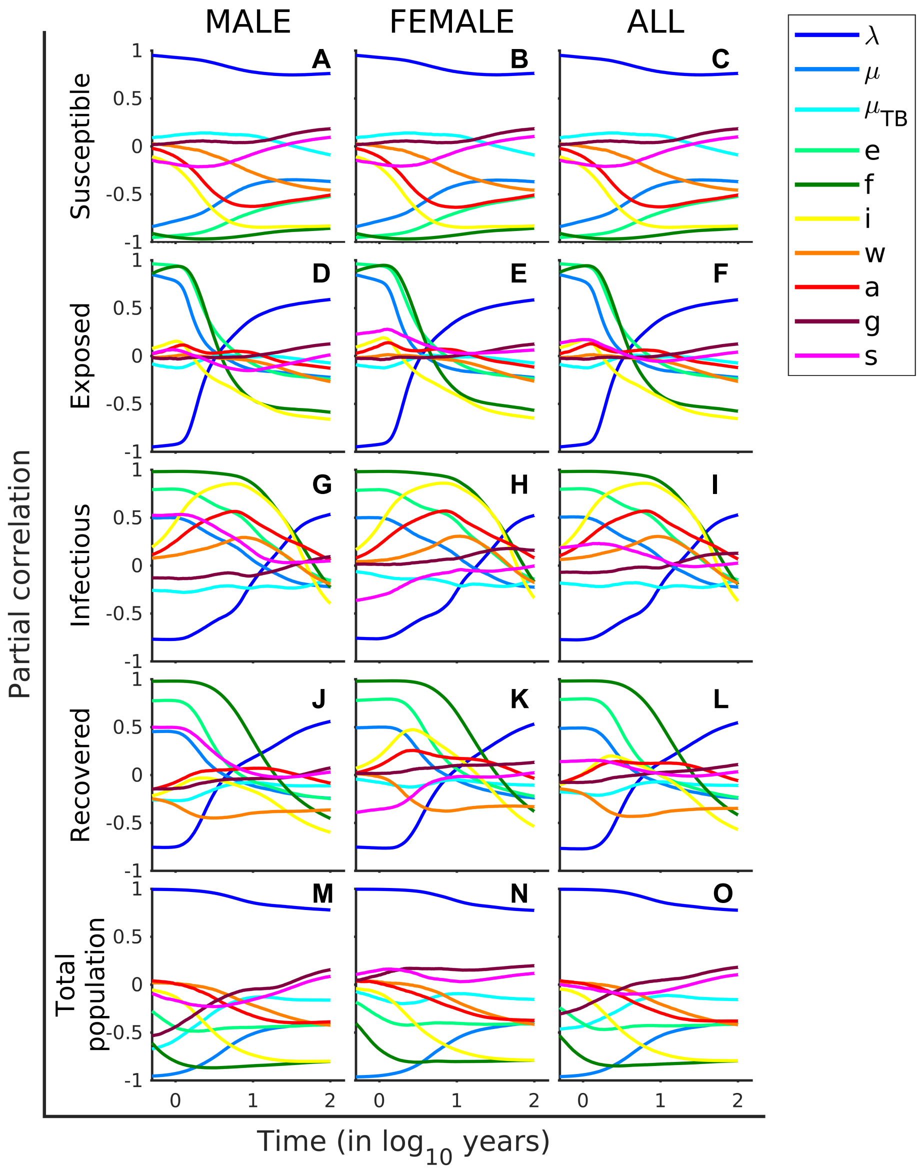


## Supplementary Figure 7

**Spearman's rank correlation coefficient between each of the parameters at each time step.** Simulations are 100 days long. Correlation coefficient is computed for: (A) Male susceptible population, (B) Female susceptible population, (C) Total susceptible population, (D) Male exposed population, (E) Female exposed population, (F) Total exposed population, (G) Male infected population, (H) Female infected population, (I) Total infected population, (J) Male recovered population, (K) Female recovered population, (L) Total recovered population, (M) Male total population, (N) Female total population, (O) Total population.

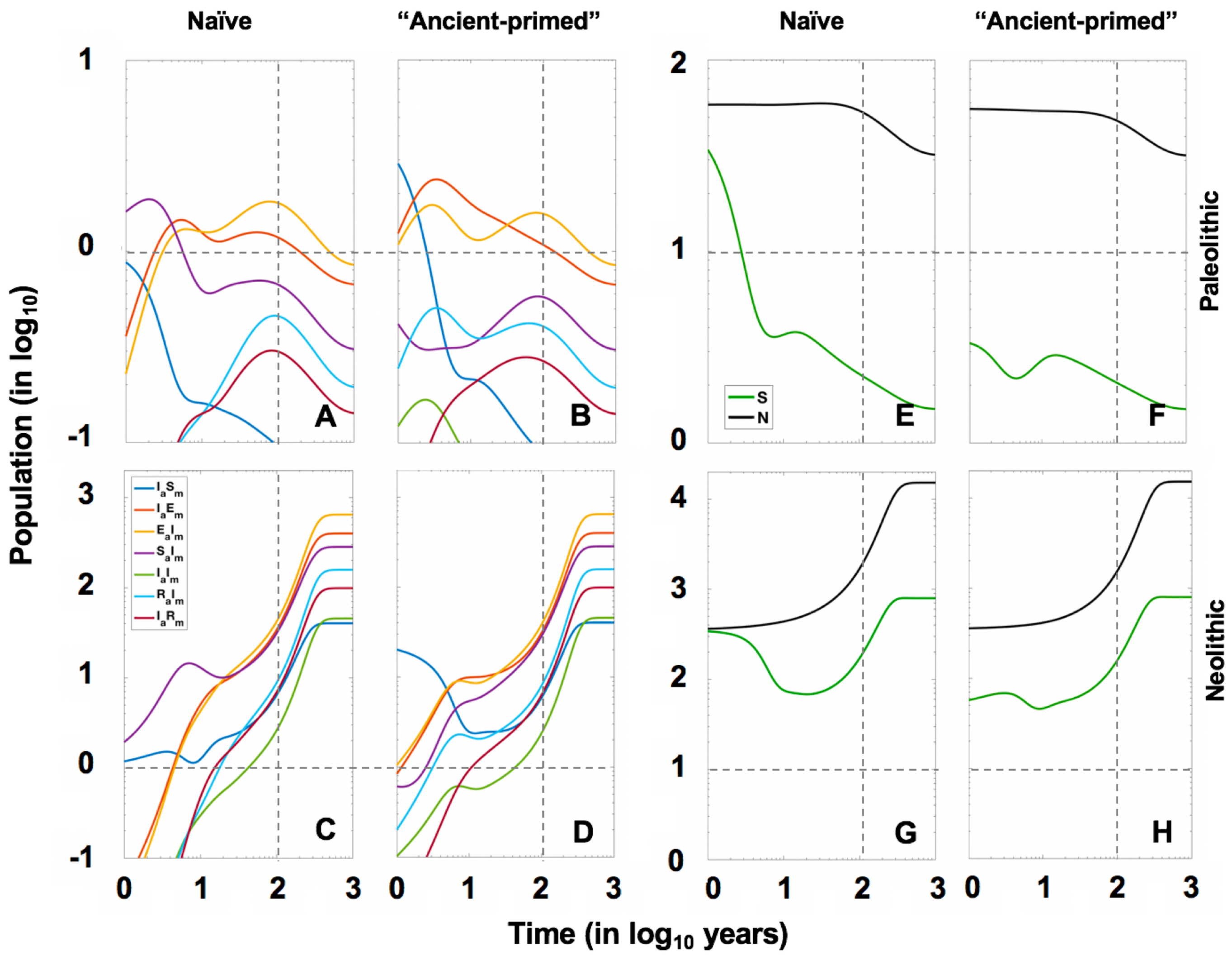


**Supplementary Figure 8**

**The replacement of "ancient" lineages by "modern" ones.**

Pictures show the evolution of the coinfection in four different circumstances, using the continuous coinfection model. Left quadrants show the evolution of the coinfection in the context of a "naive" population with two infectious males, one from each lineage, in the periods of Palaeolithic (**A**,**E**) and Neolithic (**C**,**G**). Right quadrants show the evolution of the coinfection in the context of a community where infection with an "ancient" lineage has remained stable for 100 years and one "modern" lineage infected male is introduced, in the periods of Palaeolithic (**B**,**F**) and Neolithic (**D**,**H**).

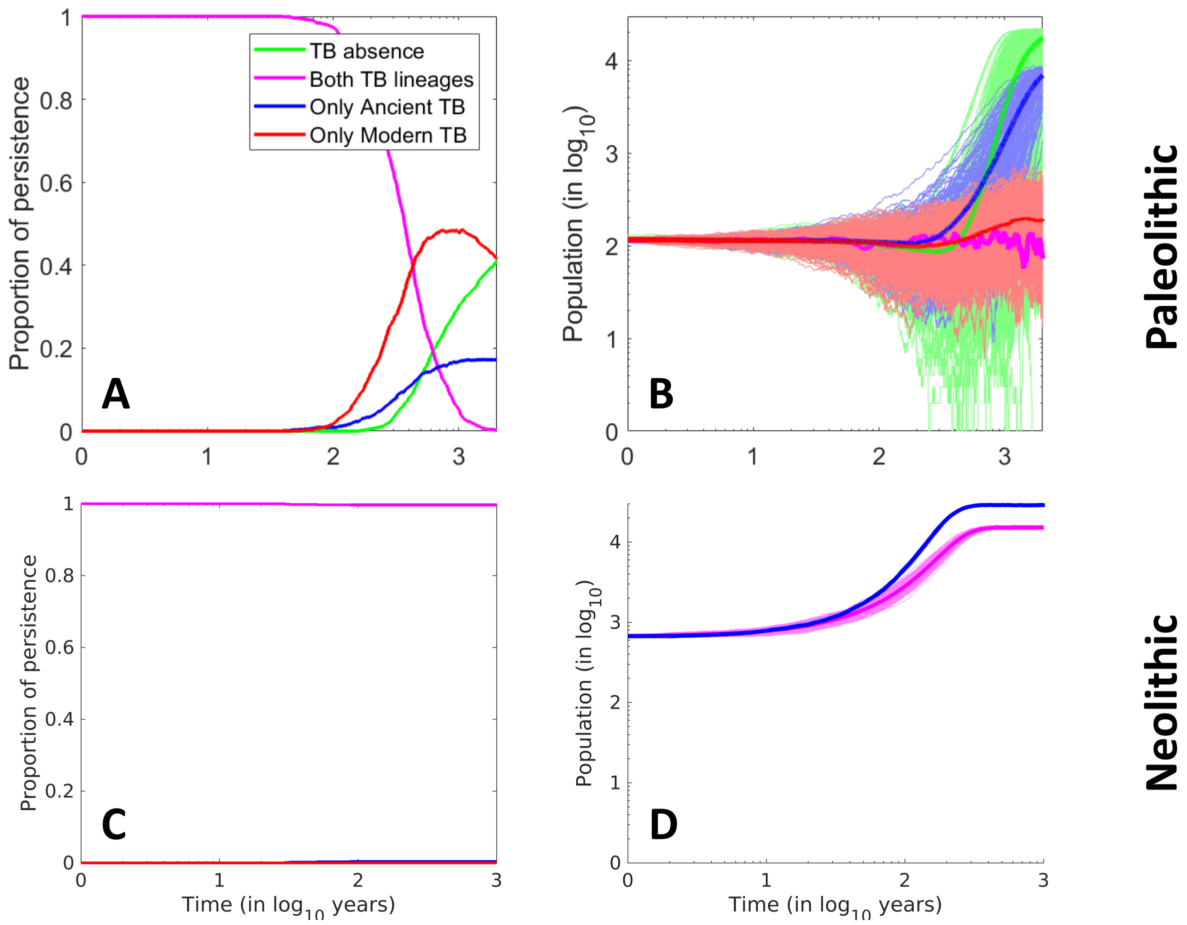


## Supplementary Figure 9

**Coexistence of "Ancient" and "Modern" lineages after the arrival of 5 sick individuals with a "Modern" strain at 10000 independent "Ancient-primed" communities.** The model is solved with the discrete resolution, with 10000 independent repetitions of each scenario (**A,B:** Palaeolithic; **C,D:** Neolithic). **A** and **C** show the **proportion of communities** with presence of both strains (magenta), only ancient (blue), only modern (red) or none of them (green) along the time. Simulated period is 2000 years for Palaeolithic scenario and 1000 years for Neolithic scenario. **B** and **D** show the evolution of **total population** in all simulations (light colours) and the mean of the communities with the same final state; in magenta, those communities whose final state is the coexistence of both lineages; in blue and red, those communities that finish with "Modern" lineage clearance and "Ancient" lineage clearance, respectively; in green, those communities that achieve complete TB clearance.

# Supplementary Table 1

**Comparison of the parameters of the basic SEIR model with those form bibliography.** See the text and original references for details.

| | Cardona et al | | Zheng et al 2014 | Blower et al 1995 | | |
|---|---|---|---|---|---|---|
| | low | high | | min | peak | max |
| **Population Growth Rate $\pi$** **$\lambda N(1 - N/Nm)$** | none | | 50/1000 | none | | |
| 0.5*λ(Nf) -λ- | 0.078 | 0.128 | none | | | |
| **Average life** | 26.5 | 33 | 50 | 25 | none | 75 |
| **Mortality (due to TB) $\mu T$** | 0.12 | 0.15 | 0.33 | 0.058 | 0.139 | 0.461 |
| **Progression rate from S to I (p)** | none | | none | 0 | 0.05 | 0.3 |
| **Fast progression from S to I (f / ff)** | 0.0825/0.09090 | 0.1031/0.1328 | 0.1 | 0.5 | 0.7 | 0.85 |
| **Slow progression from E to I (fs)** | none | | none | none | 0.5 | 0.85 |
| **Progresion rate from E to I (v)** | none | | none | 0.002556 | none | 0.00527 |
| **Progression rate from E to I (a)** | f*0.3 | | variable | none | | |
| **Regression from I to S $\delta$** | 0.1-f-a | | none | none | | |
| **Number of infected per case , e** | 10 | 20 | 10 | 3 | 7 | 13 |
| **Reduction of progression to disease in I and R -immunity- (i)** | 0.1 | | none | none | | |
| **Reduction of fast progression in reinfection in I (k)** | none | | 0.5 | none | | |
| **Increase relapse R to I (w)** | 4 | | none | none | | |
| **Rate of Relapse/y R to I (2$\omega$)** | none | | none | 0 | 0.1 | 0.03 |
| **Curation rate c** | 0.33 | | 0.25 | 0.021 | 0.058 | 0.086 |
| **Tolerance to infection s** | 55/45 | | none | none | | |
| **Tolerance to disease g** | 55/45 | | none | none | | |

## Supplementary Table 2

**Details of the natality rates explored with the model.** In blue, values used in the standard simulation. See Figure 9 for the results.

| | Paleolithic | | | | | | |
|---|---|---|---|---|---|---|---|
| **births/female** | **2,2** | **2,5** | **2,8** | **3,1** | **3,4** | **3,7** | **4** |
| fertile years | 18 | 18 | 18 | 18 | 18 | 18 | 18 |
| total/female | 0,122 | 0,139 | 0,156 | 0,172 | 0,189 | 0,206 | 0,222 |
| fertile female | 0,5 | 0,5 | 0,5 | 0,5 | 0,5 | 0,5 | 0,5 |
| $\lambda$ | **0,0611** | **0,0694** | **0,0778** | **0,0861** | **0,0944** | **0,1028** | **0,1111** |

| | Neolithic | | | | | | |
|---|---|---|---|---|---|---|---|
| **births/female** | **4** | **4,3** | **4,6** | **4,9** | **5,2** | **5,5** | **5,8** |
| fertile years | 18 | 18 | 18 | 18 | 18 | 18 | 18 |
| total/female | 0,222 | 0,239 | 0,256 | 0,272 | 0,289 | 0,306 | 0,322 |
| fertile female | 0,5 | 0,5 | 0,5 | 0,5 | 0,5 | 0,5 | 0,5 |
| $\lambda$ | **0,1111** | **0,1194** | **0,1278** | **0,1361** | **0,1444** | **0,1528** | **0,1611** |
